# Minimizing the Programming Power of Phase Change Memory by Using Graphene Nanoribbon Edge‐Contact

**DOI:** 10.1002/advs.202202222

**Published:** 2022-07-18

**Authors:** Xiujun Wang, Sannian Song, Haomin Wang, Tianqi Guo, Yuan Xue, Ruobing Wang, HuiShan Wang, Lingxiu Chen, Chengxin Jiang, Chen Chen, Zhiyuan Shi, Tianru Wu, Wenxiong Song, Sifan Zhang, Kenji Watanabe, Takashi Taniguchi, Zhitang Song, Xiaoming Xie

**Affiliations:** ^1^ State Key Laboratory of Functional Materials for Informatics Shanghai Institute of Microsystem and Information Technology Chinese Academy of Sciences 865 Changning Road Shanghai 200050 P. R. China; ^2^ Center of Materials Science and Optoelectronics Engineering University of Chinese Academy of Sciences Beijing 100049 P. R. China; ^3^ CAS Center for Excellence in Superconducting Electronics (CENSE) Shanghai 200050 P. R. China; ^4^ School of Physical Science and Technology ShanghaiTech University Shanghai 201210 P. R. China; ^5^ Research Center for Functional Materials National Institute for Materials Science 1‐1 Namiki Tsukuba 305‐0044 Japan; ^6^ International Center for Materials Nanoarchitectonics National Institute for Materials Science 1‐1 Namiki Tsukuba 305‐0044 Japan

**Keywords:** cycle endurance, edge‐contact, graphene nanoribbon, phase change cell, power consumption

## Abstract

Nonvolatile phase‐change random access memory (PCRAM) is regarded as one of the promising candidates for emerging mass storage in the era of Big Data. However, relatively high programming energy hurdles the further reduction of power consumption in PCRAM. Utilizing narrow edge‐contact of graphene can effectively reduce the active volume of phase change material in each cell, and therefore realize low‐power operation. Here, it demonstrates that the power consumption can be reduced to ≈53.7 fJ in a cell with ≈3 nm‐wide graphene nanoribbon (GNR) as edge‐contact, whose cross‐sectional area is only ≈1 nm^2^. It is found that the polarity of the bias pulse determines its cycle endurance in the asymmetric structure. If a positive bias is applied to the graphene electrode, the endurance can be extended at least one order longer than the case with a reversal of polarity. In addition, the introduction of the hexagonal boron nitride (*h*‐BN) multilayer leads to a low resistance drift and a high programming speed in a memory cell. The work represents a great technological advance for the low‐power PCRAM and can benefit in‐memory computing in the future.

## Graphical abstract

It is demonstrated that the power consumption can be reduced to ≈53.7 fJ in a phase change memory cell with ≈3 nm‐wide graphene nanoribbon (GNR) as edge‐contact, whose cross‐sectional area reaches only ≈1 nm^2^. The asymmetric structure in the device enables the finding that the polarity of bias pulse determines its cycle endurance.

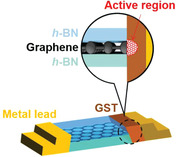

## 1 Introduction

Phase‐change random access memory (PCRAM) is a promising circuit‐building block for non‐von Neumann computing architectures by combining storage and computing functions. It is equipped with desirable properties for nonvolatile, high device density, high programming speeds, and long switching lifetime.^[^ ^1^, ^2^ ^]^ These properties are the key enablers of components for in‐memory computing in highly data‐centric applications. Therefore, in terms of materials and device structure, new phase‐change devices are highly desired to enable high throughput, area‐efficient, and energy‐efficient information processing. Both capitalizing on new phase change materials (PCMs)^[^ ^3^ ^]^ and applying an incubation field^[^ ^4^ ^]^ can lead to higher operating speed while stacking phase‐change multilayered heterostructure could extend switching lifetime.^[^ ^5^ ^]^ The greatest challenge faced by PCRAM at this moment is how to reduce its power consumption further. There are several ways to reduce the power consumption of cells: including manipulating the electrodes,^[^ ^6^, ^7^ ^]^ exploiting new materials,^[^ ^8^, ^9^ ^]^ and designing device architecture.^[^ ^10^ ^]^

The scaling on phase‐change cells not only can enable the integration of more memory devices, but also reduce the programming energy. The conventional memory cell exhibits a relatively high power consumption due to its large contact area between PCM and the heating electrode.^[^ ^11^ ^]^ The cutting‐edge technologies in the semiconductor industry can scale memory cells only down to sub‐7‐nm, but the contact area is still in tens of nm^2^. Creating a gap in carbon nanotube can decrease the active volume of phase change materials by orders and then reduce the power consumption.^[^ ^6^, ^12^, ^13^, ^14^ ^]^ Nevertheless, the technique needs extensive skills and complex processes in both nano‐gap fabrication and deposition of phase change material. Using blade electrodes is another approach to reduce the materials needed to be heated up, and then consume less energy. However, the cross‐sectional area of the memory cells with the most advanced blade electrode still exceeds 40 nm^2^.^[^ ^7^, ^15^ ^]^

Graphene is a two‐dimensional semi‐metal with a thickness of a single atom (≈0.335 nm).^[^ ^16^, ^17^ ^]^ It can make 1D electrical contact with other materials by edge‐contact.^[^ ^18^, ^19^ ^]^ In addition, graphene is chemically inert and thermally stable, with a current‐carrying capacity of more than 10^9^ amps per square centimeter.^[^ ^20^, ^21^ ^]^ The merits are highly desired in electrodes for phase change programming. Therefore, graphene can serve as the thinnest blade‐electrode for PCRAM cells in nature. Recently, lateral PCRAM cells with patterned GNR electrodes were demonstrated at Stanford University.^[^ ^22^ ^]^ However, the power consumption of the cells is still one order of magnitude greater than those with CNT electrodes due to the challenges in process control over the quality of both GNR–GST interface and GST material. Encapsulation of GNRs with hexagonal boron nitride (*h*‐BN) flakes can preserve their intrinsic properties by keeping them from the ambient environment even under programming operation. Besides, the *h*‐BN flake in a suitable thickness can serve as a “heat sink” in programming because of its high in‐plane thermal conductivity.^[^ ^23^, ^24^ ^]^ Bringing both GNR and *h*‐BN flakes to contact PCMs with their edges builds up a new design for the structure of PCRAM cells.

In this report, we adapt the edges of graphene nanostructure as blade‐contact to memory cells. Here, Ge_2_Sb_2_Te_5_ (GST) is selected as PCM as it has been widely used in PCRAM and extensively studied. The dimensions of the cells are defined by the width of edge‐contact of graphene (≈3 nm to 2 µm). As the edge is the location with the highest E‐field and Joule heating in graphene, switching of PCMs occurs only near the edge‐contacts. It is found that the switching speed could reach 5 ns in a memory cell with graphene edge‐contact. Its cycle endurance reaches about 6 × 10^6^ if a proper bias polarity is applied. The power consumption of the memory cell decreases to ≈53.7 fJ when the contact width decreases to ≈3 nm. The corresponding cross‐sectional area of the edge‐contact is only ≈1 nm^2^. Its operation duration is close to 3 × 10^5^, which allows reliable iterative programming operations. In addition, a D flip‐flop prototype working under 2.5 MHz was demonstrated in a memory cell with graphene nanoribbon (GNR) edgecontact. The detailed process for fabrication of a memory cell with edge‐contact is described in Experimental Section and Supporting Information (Figure S1, Supporting Information).

## 2 Results and Discussion

**Figure 1:**
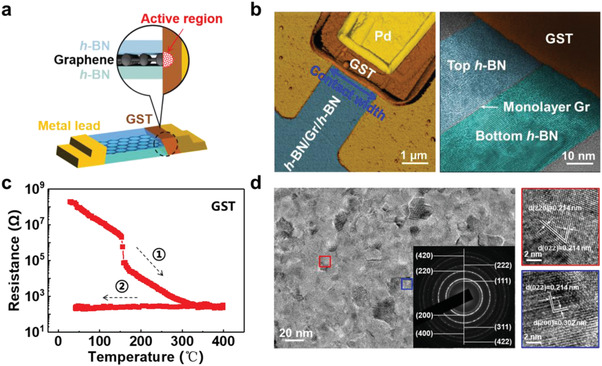
Memory cell with graphene edge‐contact. a) Schematic of the cell. b) AFM image of a memory cell where palladium (Pd), GST, and *h*‐BN/Gr/*h*‐BN are painted with gold, brown and cerulean, respectively. The scale bar is 1 µm. The HRTEM image on the right shows a cross‐sectional view of the edge‐contact, the scale bar is 10 nm. c) The resistance variation of a 60 nm‐thick GST film in a cycle of annealing. The heating and cooling rates are ≈20 and ≈100 °C min^−1^, respectively. d) TEM investigation on another GST film annealed at ≈260 °C, and the insert shows the corresponding SAED pattern. Zoom‐in views on two specific crystal grains framed are given on the right.

**Figure** **1**a shows a schematic of a memory cell by using graphene edge as 1D contact. Its AFM image is given on the left of Figure 1b. The width of edge‐contact is about 2 µm. A high‐resolution transmission electron microscopy (HRTEM) image shows a cross‐sectional view of edge‐contact on the right of Figure 1b. The blue and green areas represent the capping and bottom layer of *h*‐BN, respectively. The grey contrast comes from their different lattice orientations. The position of the graphene layer is also pointed out in Figure 1b. Figure 1c shows the resistance‐temperature relationship of GST, which exhibits a crystallization transition at the temperature of ≈155.7 °C. The data retention of the RESET state in GST cells is expected to be ≈80.2 °C for 10 years by Arrhenius fitting (Figure S2, Supporting Information). As shown in Figure 1d, the selected area electron diffraction (SAED) pattern of the GST film exhibits that the nanocrystal grains exhibit a face‐centered cubic structure, which was further confirmed with HRTEM images.

**Figure 2:**
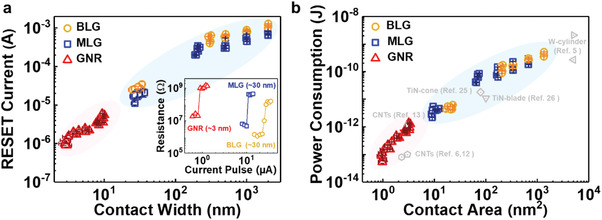
Scaling trend of power consumption. a) RESET current versus width of edge‐contact in memory cells. Insert shows RESET process driven by current pulses in the memory cells with different edge‐contact. b) Power consumption as a function of the contact area. The yellow circle, blue square, and red triangle represent the results from the 74 cells with edge‐contact of BLG (26 pieces), MLG (29 pieces), and GNR (19 pieces), respectively. The other symbols in gray are data on power consumption adapted from literature.^[^ ^5^, ^6^, ^12^, ^13^, ^25^, ^26^ ^]^

Bilayer graphene (BLG), monolayer graphene (MLG), and GNR of different widths are used as the edge‐contact of memory cells. The narrowest pattern of graphene defined by lithography is a width of ≈30 nm. In order to narrow the contact further, GNRs embedded in *h*‐BN^[^ ^27^ ^]^ were used as edge‐contact to PCMs. The width of GNRs used here is less than 10 nm. As the SET energy *E*_SET_ is almost three orders lower than the RESET energy *E*_RESET_, it is always ignored when calculating the power consumption of memory cells.^[^ ^28^ ^]^ Before measurement, the memory cells were annealed at ≈260 °C in order to set the initial state of GST ready for the test to a low resistance state (LRS, also known as ON state). Subsequently, a series of current pulses with a fixed duration (100 ns) were sourced to the cell via graphene edge‐contact with the magnitude of the pulses gradually increasing. Simultaneously, the resistances of memory cells were recorded. And the results are shown in the inset of **Figure** **2**a. The action to set the cell from LRS to a high resistance state (HRS, also called OFF state) is marked as RESET, this switching is corresponding to the quick melting and quenching of GST, turning the material into the amorphous phase. Here, the minimum current pulse switching the cell to HRS is defined as RESET current. (More results about RESET current are shown in Figure S3, Supporting Information) The variation of RESET current with respect to the width of edge‐contact is plotted in Figure 2a. It is found that RESET current monotonously decreases with the shrink of contact width, while the cell resistance measured increases. It is also found that the RESET current of memory cells with BLG contact is almost twice that of cells with MLG contact in the same width. The RESET current measured is ≈11 µA in the cell with ≈30 nm‐wide edge‐contact of MLG. And the RESET current in a pulse of 100 ns obtained is ≈0.9 µA in a memory cell with a ≈3 nm‐wide GNR edge‐contact.

Subsequently, the contact‐area dependence of power consumption ($E=\int_{0}^{\Delta t}IVdt$) of each memory cell is extracted, where *I* represents RESET current, *V* is RESET voltage, and Δ*t* is a time symbol for pulse duration. Transient RESET voltage measured is recorded once the cell completes the RESET action. The relationship of power consumption versus contact area in memory cells is plotted in Figure 2b. The power consumption of low‐power PCRAMs reported in literature was also included for comparison. It is found that the power consumption decreases with the contact area. The power consumption decreases to ≈53.7 fJ for the cell with ≈3 nm‐wide GNR edge‐contact (The measurement, extraction, and calculation method were detailed in Figure S4, Supporting Information). The value is obviously smaller than those reported in the literature, enabled by the tiny amount of GST addressed with the GNR electrode. The decrease in power consumption attributes to the shrinking of the contact area between the electrodes and GST.

**Figure 3:**
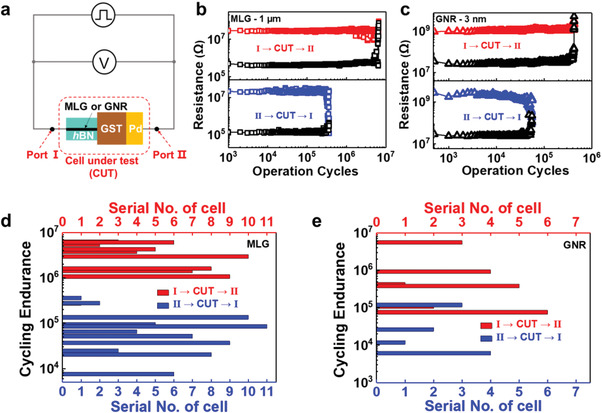
Endurance dependence on voltage polarity in the cells with an asymmetric structure. a) Schematic of the cell structure and measurement layout. b) Endurance test in the cells with MLG edge‐contact. The data shown in the upper diagram were obtained with 1.5 V/100 ns SET pulse and 2.5 V/100 ns RESET pulse in a cell (*#MLG 74*) with ≈1 µm wide MLG edge‐contact, while those shown in the bottom diagram were taken with 1.5 V/100 ns SET pulse and 2.8 V/100 ns RESET pulse in another cell (*#MLG 55*) in a similar configuration. c) Endurance investigation of a cell with ≈3 nm wide GNR edge‐contact. The results in the upper diagram were obtained with 0.5 V/100 ns SET pulse and 1 V/100 ns RESET pulse (*#GNR 103*), while those shown in the bottom diagram were taken with 0.6 V/100 ns SET pulse and 1.2 V/100 ns RESET pulse in another cell (*#GNR 108*) in a similar configuration. As shown in (b) and (c), voltage pulse applied from Port I→CUT→II leads to long endurance and “Stuck RESET” failure as shown in the cell while applying a voltage pulse from Port II to I through CUT results in short endurance and failure in a low resistance state (“Stuck SET”) in both cells. d,e) Statistics on the cycle endurance for MLG and GNR edge‐contact memory cells, respectively. It is found that the cycle endurance exhibits obvious the polarity of bias pulse in the asymmetric devices. If a positive bias was applied to the graphene electrode, the endurance could be extended at least one order longer than the case with a reversal of polarity.

Cycle endurance is extensively investigated in the cells with edge‐contact. The voltage for the operation of “Read” always is kept at 0.1 V unless otherwise noted. The cells using MLG/GNR as edge‐contact are physically asymmetric in their device structure. The asymmetry in device structure has a significant influence on the device endurance under different voltage polarities. A schematic of the cell and measurement setup is shown in **Figure** **3**a. The results of cycle‐endurance measured in ≈1 µm MLG edge‐contact cells and GNR edge‐contact cells in ≈3 nm width are plotted in Figure 3b,c, respectively. The memory cell with ≈1 µm MLG edge‐contact (*#MLG 74*) exhibits an endurance close to 6 × 10^6^ (Figure 3b), and the ≈3 nm GNR edge‐contact memory cell (*#MLG 103*) shows an endurance up to 3 × 10^5^ when the voltage pulse is applied from Port I to II (Figure 3c). Meanwhile, when a pulse voltage was applied from Port II to I through CUT, the endurances become just around 10^5^, as shown in Figure 3b,c. It is also noted that the voltage polarity has a strong effect on failure modes. When voltage pulse was applied from Port I → CUT → II, the cell fails in a high resistance state (“Stuck RESET”). Reversal of voltage polarity always leads to the failure of “Stuck SET”.

A systematic statistical investigation on cycle endurance was also carried out, and the results are shown in Figure 3d,e, respectively. As shown in Figure 3d,e a relatively long endurance can be achieved in most of the cells with edge‐contact if the voltage pulse was applied from Port I to II through CUT. Cells applied with voltage pulse direction (Port I → CUT → II) are all found to fail in “Stuck RESET”, while those in the reversal of voltage polarity fail in “Stuck SET” (See Figure S6, Supporting Information). Figure S7, Supporting Information shows a cross‐sectional TEM image of the cell after “Stuck RESET” failure. The void, which is profiled by the white dash line in Figure S7, Supporting Information, is found at the contact between graphene‐edge and GST. The appearance of a void is normally regarded as the signature for “Stuck RESET”.

A cross‐sectional TEM image and elemental EDS maps of the cell with “Stuck SET” failure after the endurance test was shown in Figure S8, Supporting Information. As shown in Figure S8f–h, Supporting Information, the signals of Ge, Sb, and Te elements extended into the Van der Waals (vdW) interface of *h*‐BN/graphene by emigration. As shown in Figure S8i, Supporting Information, the counts of Ge elements are slightly more than Sb and Te in the active region (colored with red). The composition of GST in the gray region of Figure S8i, Supporting Information is similar to the composition of GST (Figure S8j, Supporting Information) in the inactive region marked in Figure S8a, Supporting Information. This indicates that the size of the active region is ∼10 nm. When the voltage pulse was applied from Port II → CUT → I, elements in GST emigrate into the interlayers of graphene/*h*‐BN under the electric and thermal fields, and then form a small confined active region. In this tiny region, the element segregation of the GST under the cycle test greatly degrades the cycle endurance with “Stuck SET” failure.

In addition, thermoelectric properties (Peltier and Thomson effects) may be one important factor for memory cells because thermoelectric heating can significantly influence the temperature distribution in the memory cells.^[^ ^29^ ^]^ If thermoelectric heating exists during programming in PCM cells, it could be a supplement to Joule heating. It is possible to qualitatively achieve some signature of the thermoelectric effects by comparing the difference of RESET voltage under different polarities in the same device.^[^ ^30^ ^]^ In order to compare the effect of the voltage polarity on the RESET voltage, we carried out the statistical analysis on the amplitudes of voltage pulse under different polarities in the same memory cell without taking cycle endurance measurements in some devices. As shown in Figure S9b, Supporting Information, little difference was observed in the RESET voltages of the two polarities. It indicates that thermoelectric heating may not be pronounced during programming in the cells with graphene.

The resistance drift was also measured in the cells with graphene edge‐contact (Figure S10, Supporting Information), and the coefficient of resistance drift is found to be close to 0.02, which is significantly smaller than that in memory cells with cylinder electrode (*v = ≈*0.1).^[^ ^31^ ^]^ In order to explore the main possible reason for the small drift of resistance, we fabricated some memory cells addressed with graphene edge‐contact. The difference is that *h*‐BN multilayers were damaged by N_2_ plasma before assembling vdW heterostructures. As shown in Table S2, Supporting Information, the substrate with high thermal conductivity is beneficial to improve the resistance stability of amorphous GST while it may also lead to an increase in power consumption, and therefore sometimes shorten the cycle endurance. The small resistance drift, we believe, may be related to the extremely high in‐plane thermal conductivity of graphene^[^ ^32^ ^]^ and *h*‐BN flakes,^[^ ^23^, ^24^ ^]^ contacting the active region of phase change. In general, a high thermal conductivity should lead to a faster cooling rate and thus more unrelaxed local structures. And then, reducing the thermal relaxation of GST in the RESET programming process will enhance the resistance drift because the unrelaxed local structures will gradually relax after programming and thereby lead to resistance drift. The normal knowledge fails to provide an explanation on our experiment results. The falling time of programming pulses is an important factor to tune the resistance drift by affecting the structural relaxation of the active region.

In order to verify the influence of falling time on the resistance drift, we set the falling time to 3, 4, 5, and 6 ns, respectively. “3 ns” is the minimum value that our test system can be set to. However, we did not find obvious dependence of resistance drift on the falling time. A longer falling time than 6 ns always spoils the amorphous state of the device. In this case, it looks very difficult to achieve a conclusive result about the influence of falling time of voltage pulse on resistance drift at this moment.

It is indeed an interesting issue that is really worth further investigation. Here, we would like to give two possible reasons for the small resistance drift: 1) Graphene edge‐contact reduce the volume of the active region, and the small active region normally leads to less stress together with the high thermal conductivity of graphene and *h*‐BN. 2) The weak vdW interactions between GST and *h*‐BN or GNR could cause full relaxation of amorphous GST.^[^ ^33^ ^]^ In contrast, the defective *h*‐BN or SiO_2_ results in more reactive bonds whose reaction and relaxation may lead to a higher resistance drift.

As the nonvolatile memory cells can perform the function of resistive switching, we demonstrated the logic function of D latch based on the memory cells with edge‐contact. The results are shown in Figure S11, Supporting Information. Figure S11a, Supporting Information displays the logic function of the D latch. Input signals can modify the magnitude of output resistance. It is always expressed as the switching of logic states in the cell. Here, “D”, “Q”, and “Q*” denote input, initial state, and subsequent state, respectively. Both pulses of high voltage (2.5 V) and HRS are defined as logical “1”, while the pulses of low voltage (1.5 V) and LRS are defined as logical “0”. As shown in Figure S11a, Supporting Information, if no voltage pulse is applied (D = “zero input”), the subsequent state (Q*) remains the initial state (Q). When a voltage pulse in 1.5 V (D = logical “0”) is sourced, the cell switches from the HRS to LRS. Similarly, a voltage pulse of higher amplitude (2.5 V) (D = logical “1”) will switch the cell from the LRS to HRS. The voltage for reading the cell resistance always remains less than 0.1 V unless otherwise noted. The corresponding truth table and the logic diagram are shown in Figure S11b,c, Supporting Information, respectively. In this design, Q/Q* and input D share the same pin, and they are separated by a switching circuit. The resistance state of the cell (also known as Q/Q*) can be read if the 1T is open. A small voltage (≈0.1 V) which is applied to read the resistance state of the memory cell does not affect the state of the cell.

**Figure 4:**
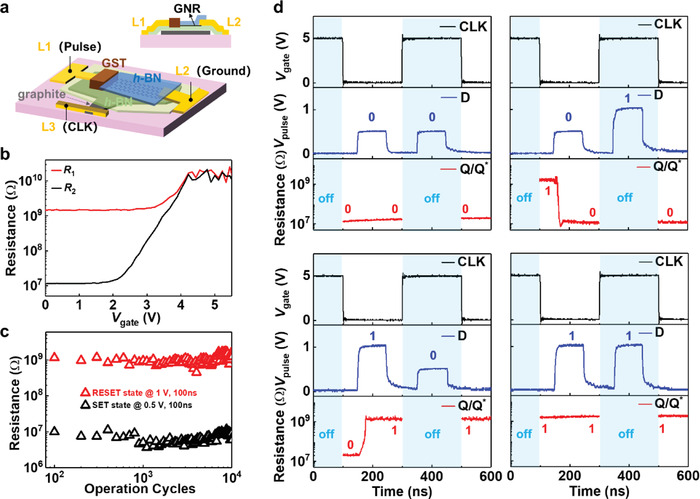
A prototype of the D flip‐flop made from a memory cell with GNR edge‐contact. a) Schematics of the prototype and the measurement setup. b) The cell resistance versus gate voltage (*V*_gate_) at 300 K. c) Cycle endurance of the memory cell. Both SET (0.5 V)/RESET (1 V) signals are in a pulse width of 100 ns. d) Demonstration of logic functions in the D flip‐flop.

On this basis, we prepared a D flip‐flop made from a memory cell with GNR edge‐contact. The fabrication process for the D flip‐flop based on the memory cell with edge‐contact of GNR is shown in Figure S10, Supporting Information. **Figure** **4**a shows a schematic and measurement setup for the D flip‐flop. During operation, L1(connected to GST) and L3 (connected to the graphite which serves as the back‐gate) are selected as the input (D) and the clock signal (CLK), whereas L2 (connected to GNR) is grounded. To investigate the gate tunability of the cell with GNR edge‐contact, a small lateral voltage (0.1 V) was applied through L1 and L2 to measure the resistance by sweeping *V*_gate_ from 0 V to 5.5 V. Figure 4b shows the resistance of the cell versus *V*_gate_ at 300 K. The red and black line were measured when the memory cell in HRS and LRS, respectively. It is found that the channel of the cell with GNR electrode is in ON‐state when *V*_gate_ = 0 V and OFF‐state when *V*_gate_ = 5 V (see the *R*–*V*_gate_ curve in Figure S13a, Supporting Information, the GNR transistor exhibits *R*_H_ (*V*_gate_ = 0 V)/*R*_L_ (*V*_gate_ = 5 V) ≈10^5^ at 300 K).

In order to test its cycle endurance, we set 0.5 V/1 V as SET/RESET voltage with a pulse width of 100 ns to complete the reversible switch from HRS (≈10^9^ Ω) to LRS (≈10^7^ Ω), as shown in Figure 4c. The memory cell shows a cycle endurance of more than 10^4^.

Figure 4d shows a dynamic logic function of the D flip‐flop under a clock (CLK) signal of 2.5 MHz. The test conditions are the same as that in Figure 4c. The input D in a voltage pulse of 0.5 V (1 V) is regarded as logical “0” (“1”). Q and Q^⁎^ represent the initial and subsequent resistance state of the memory cell, respectively. The LRS of the cell means logical “0” while HRS in the cell is considered as logical “1”. When CLK is in 0 V (logical “1”), Q^⁎^ becomes logical “0” or “1” regardless of the initial resistance state, by following D. However, when CLK is in 5 V, Q^⁎^ will not change but remains in its initial state of Q. The transient change of resistance can be measured directly by an oscilloscope, the details of measurement are shown in Figure S14, Supporting Information. It is noted that the switch between two states takes ≈20 ns, greatly shorter than 100 ns of the input pulse. This indicates that the excrescent electrical energy converts to the Joule heat without causing amorphous–crystalline transitions. The corresponding truth table is shown in Figure S15b, Supporting Information. D flip‐flop normally takes lots of transistors to implement in the traditional CMOS technology. Here, the memory cell with GNR edge‐contact realized the logic function of a D flip‐flop, which just is composed of PCMs and a GNR channel. In addition, the D flip‐flop is the capability of nonvolatile memory. It could be used to produce low‐power arithmetic/logic units in nonvolatile random‐access memories.

## 3 Conclusion

Scaling PCRAM cells to low dimensions could produce more new physics to boost their performance if the GST was engineered or changed to other PCMs. A cell with edge‐contact achieves a SET speed of ≈6 ns at ≈0.45 V, and it exhibits an ultra‐low power consumption of ≈53.7 fJ in a high endurance of 3 × 10^5^ when the width of contact approaches 3 nm. The power consumption of each cell is almost several orders of magnitude lower than that in the state of art PCRAM cells,^[^ ^34^, ^35^, ^36^ ^]^ and even half lower than that in the cell addressed by a CNT gap.^[^ ^6^ ^]^ The edge‐contact geometry in PCRAM devices could help explore the dynamic switching process of phase‐change material at the atomic level and engineer different materials, and then improve their electrical performance. The asymmetric structure of the memory cell with edge‐contact enables a great enhancement of cycle endurance if the proper polarity of bias pulse was applied. In addition, the introduction of the *h*‐BN multilayer leads to a low resistance drift and a high programming speed in a memory cell.

From an application point of view, the performance of PCRAM cells made from graphene edge‐contact allows them to serve as nonvolatile DRAM^[^ ^37^ ^]^ in the future if their endurance is improved further via engineering PCMs or device configuration. The prototype of the D flip‐flop with GNR edge‐contact (Figure S12, Supporting Information) shows obvious practical advantages in energy efficiency compared with PCRAM cells made from silicon transistors. A narrow GNR not only brings the blade contact to PCM but also serves as the channel of a field‐effect transistor with a high on/off ratio. Phase change devices with edge‐contact of GNRs have shown their potential as the building blocks of unconventional computing architectures to bypass the von Neumann bottleneck^[^ ^38^, ^39^ ^]^ and overcome the limits of Dennard scaling.^[^ ^40^ ^]^

## 4 Experimental Section

### Device Fabrication

Both *h*‐BN and graphene flakes were prepared from their bulks by mechanical exfoliation.^[^ ^41^ ^]^ A pick‐up technique similar to the literature^[^ ^42^ ^]^ was adapted to sandwich graphene in between two *h*‐BN layers. And a mask of PMMA resistance was defined by lithography on the capping *h*‐BN surface. The regions of the heterostructure outside of the mask were then etched away to expose the edge of graphene. Pd electrodes in a thickness of 50 nm were deposited by a magnetron sputter. After another small window was opened to expose the edge of graphene, the GST material was deposited by the magnetron sputter. A diagram of the preparation process is shown in Figure S1, Supporting Information.

In order to verify the quality of the electrical contact formed between the graphene edge and Pd, the contact resistances *R*_c_ were extracted by the transfer length method (TLM). The results are shown in Figure S16, Supporting Information, it is found that the *R*_c_ is ≈600 Ω µm when *V*_gate_ = 0 V at 300 K and varies a little under different temperatures.

The GNRs were grown by chemical vapor deposition.^[^ ^27^ ^]^ The fabrication for the D flip‐flop based on the memory cell with edge‐contact of GNR is shown in Figure S12a, Supporting Information. A flake of graphite was peeled onto the silicon substrate with 300 nm SiO_2_. After that, *h*‐BN, which was in suitable thickness (≈50 to 60 nm), was then transferred onto the graphite. And then a *h*‐BN flake with embedded GNRs was transferred onto the *h*‐BN flake. After that, two metal leads were applied to GNRs while a metal lead was connected to graphite. Subsequently, a *h*‐BN layer with a thickness of ≈20 nm covers the GNR devices and acts as a protective layer. Finally, a window was opened by etching and then the GST with ≈30 nm‐thick was deposited into the window to connect the metal lead and GNR.

### GST Deposition

GST films were deposited by using a sputtering system (ULVAC JEOL 7800F). The deposition rate was kept at ≈0.6 Å s^−1^ at 20 W RF power to minimize the damage to the GNR or graphene.

### Electrical Measurement

Before electrical measurement, the memory devices were annealed at ≈260 °C for 3 min in nitrogen flow using a RTP. Electrical measurements were performed with the combination of a Keithley 4200 semiconductor characterization system (SCS), an arbitrary waveform generator (Tektronix AWG5002B), and two Source Meters (Keithley 2400‐C and 2602A). The resistance of the device (also known as the state of the device) was measured by applying a DC bias of 0.1 V, which would not change the state of the device. And the transient voltages were recorded by an oscilloscope (Tektronix MDO3032).

### Raman Spectroscopy Characterization

The number of layers for graphene was determined by Raman spectroscopy with an excitation line of 532 nm, and the power of the laser was kept at less than 1 mW.

### Atomic Force Microscopy (AFM)

The thickness of graphite and *h*‐BN was measured by an AFM (Dimension Icon, Bruker) in tapping mode. And the GNRs were located by AFM before encapsulating by a capping layer of *h*‐BN.

### TEM and STEM Characterization

The film of GST after annealing at 260 °C for 3 min was characterized by TEM (JEOL 2100F). And the cross‐sectional edge‐contact and the width of GNRs were performed in a double Cs‐corrected TEM/STEM (JEM‐ARM300F, JEOL) instrument operated at 80 kV.

### Simulation Calculation

In order to study the heat distribution in phase‐change memory cells, a comprehensive 3D finite element model was used to analyze the heat distribution in GST, and the classical molecular dynamics simulations were used to analyze the heat transport in the graphene and *h*‐BN multidimensional heterostructure. The details are shown in Figure S17, Supporting Information.

## Conflict of Interest

The authors declare no conflict of interest.

## Author Contributions

X.W. and S.S. contributed equally to this work. H.W. conceived and designed the research. H.W., Z.S. and X.X. supervised the research work. X.W. fabricated the devices and carried out electrical measurements. L.C., H.S.W., C.C. and C.J. prepared the GNRs. S.S., T.G., Y.X. and R.W. performed the sputtering deposition of GST. C.C. and C.J. performed AFM measurements. T.G. and Y.X. carried out the TEM measurements. S.Z. and W.S. performed the modeling calculation of heat distribution for memory cell with graphene edge‐contact. H.W., S.S. and X.W. analyzed the experimental data and designed the figures. H.W., X.W. and S.S. co‐wrote the manuscript, and all authors contributed to critical discussions of the manuscript.

## Supporting information

Supporting Information

## Data Availability

The data that support the findings of this study are available in the supplementary material of this article.
