# Supplementary material for: Minimizing the Programming Power of Phase Change Memory by Using Graphene Nanoribbon Edge‐Contact
Source: Adv Sci (Weinh). 2022 Jul 18;9(25):2202222. doi: 10.1002/advs.202202222 (PMC9443440; doi:10.1002/advs.202202222)
Supplement: Supplementary file 1 — Supporting Information [file ADVS-9-2202222-s001.pdf]

ADVANCED SCIENCE
Open Access

Supporting Information

for *Adv. Sci.*, DOI 10.1002/advs.202202222

Minimizing the Programming Power of Phase Change Memory by Using Graphene Nanoribbon Edge-Contact

Xiujun Wang, Sannian Song, Haomin Wang, Tianqi Guo, Yuan Xue, Ruobing Wang, HuiShan Wang, Lingxiu Chen, Chengxin Jiang, Chen Chen, Zhiyuan Shi, Tianru Wu, Wenxiong Song, Sifan Zhang, Kenji Watanabe, Takashi Taniguchi, Zhitang Song* and Xiaoming Xie*

Supplementary Information

Minimizing the programming power of phase change memory by using graphene nanoribbon edge-contact

Xiujun Wang[1,2,3†], Sannian Song[1,2†], Haomin Wang[1,2,3*], Tianqi Guo[1], Yuan Xue[1,2], Ruobing Wang[1,2], HuiShan Wang[1,2,3], Lingxiu Chen[1,3], Chengxin Jiang[1,3,4], Chen Chen[1,2,3], Zhiyuan Shi[1,3], Tianru Wu[1,2,3], Wenxiong Song[1,2], Sifan Zhang[1], Kenji Watanabe[5], Takashi Taniguchi[6], Zhitang Song[1,2*], Xiaoming Xie[1,2,3,4]

[1] State Key Laboratory of Functional Materials for Informatics, Shanghai Institute of Microsystem and Information Technology, Chinese Academy of Sciences, 865 Changning Road, Shanghai 200050, P.R. China.

[2] Center of Materials Science and Optoelectronics Engineering, University of Chinese Academy of Sciences, Beijing 100049, P.R. China.

[3] CAS Center for Excellence in Superconducting Electronics (CENSE) Shanghai 200050, P. R. China.

[4] School of Physical Science and Technology, ShanghaiTech University, Shanghai 201210, P. R. China.

[5] Research Center for Functional Materials, National Institute for Materials Science, 1-1 Namiki, Tsukuba 305-0044, Japan.

[6] International Center for Materials Nanoarchitectonics, National Institute for Materials Science, 1-1 Namiki, Tsukuba 305-0044, Japan.

[†] These authors contribute equally to this work.
[*] Electronic mail: hmwang@mail.sim.ac.cn, ztsong@mail.sim.ac.cn

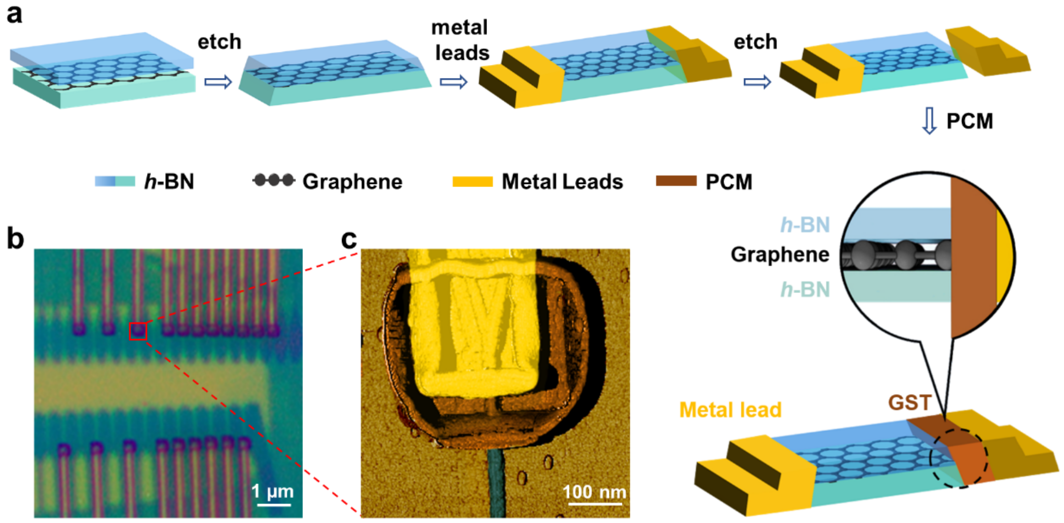

Figure S1. Making one dimensional electrical contact. a) Schematic of the fabrication process for memory cells with edge-contact. **b**) Optical and **c**) AFM image of memory cells with graphene electrode in a width of ~30 nm.

In order to achieve one dimensional edge-contact between graphene and phase-change material (PCM), we use pick-up technology [1] to sandwich graphene in-between two *h*-BN layers as shown in Figure S1a. And a mask of PMMA resist is defined by lithography on the capping *h*-BN surface. The regions of hetero-structure outside of the mask are then etched away to expose the edge of graphene. Pd electrodes in thickness of ~50 nm are deposited by magnetron sputter. After another small window is opened to expose the edge of graphene, the GST material is deposited by magnetron sputter. Figure S1b shows the optical image of memory cells with MLG edge-contact, the width is ~30 nm. And its AFM image is given at Figure S1c.

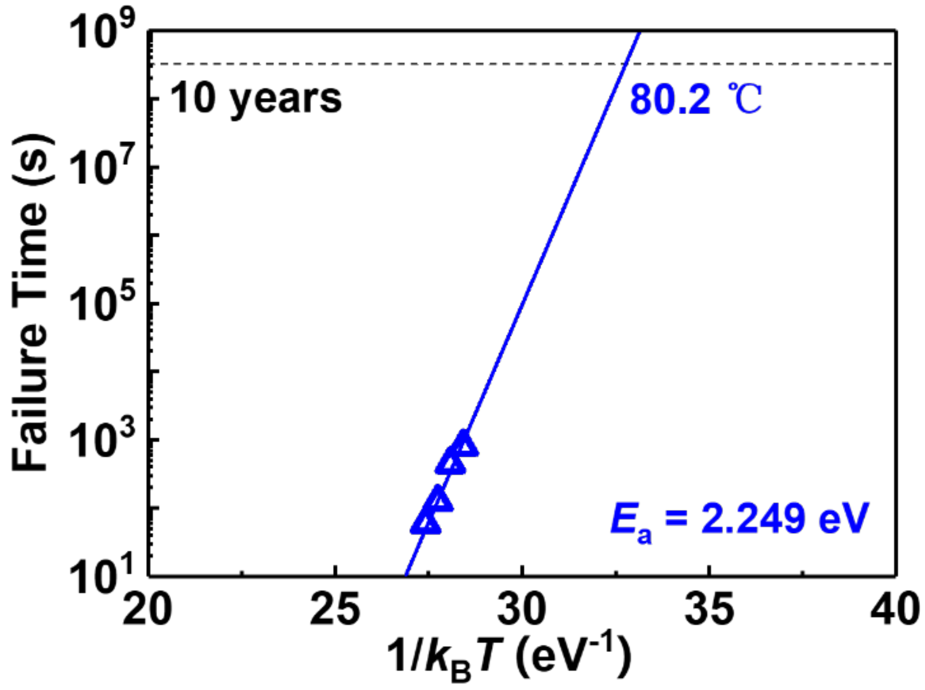

Figure S2. Failure time *versus* reciprocal temperature under isothermal heating for GST.

The relationship of failure time versus reciprocal temperature was fitted by using the Arrhenius equation $t = \tau \exp(E_a/k_BT)$, where t is the time to failure when sheet resistance of amorphous film, at a certain isothermal heating temperature, falls to half of its initial magnitude, τ is a proportionality constant, E_a is the activation energy, and k_B is the Boltzmann constant.

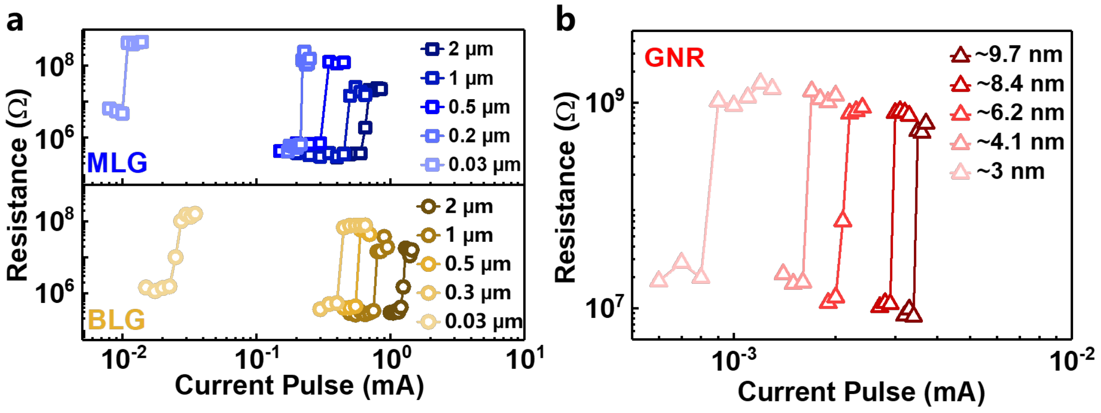

Figure S3. Resistance of memory cells *versus* current pulse magnitude in a) the memory cells with edge-contact of MLG (blue) and BLG (yellow) in different contact widths and **b**) the memory cells with edge-contact of GNR in different contact widths. The width of the RESET pulses are 100 ns.

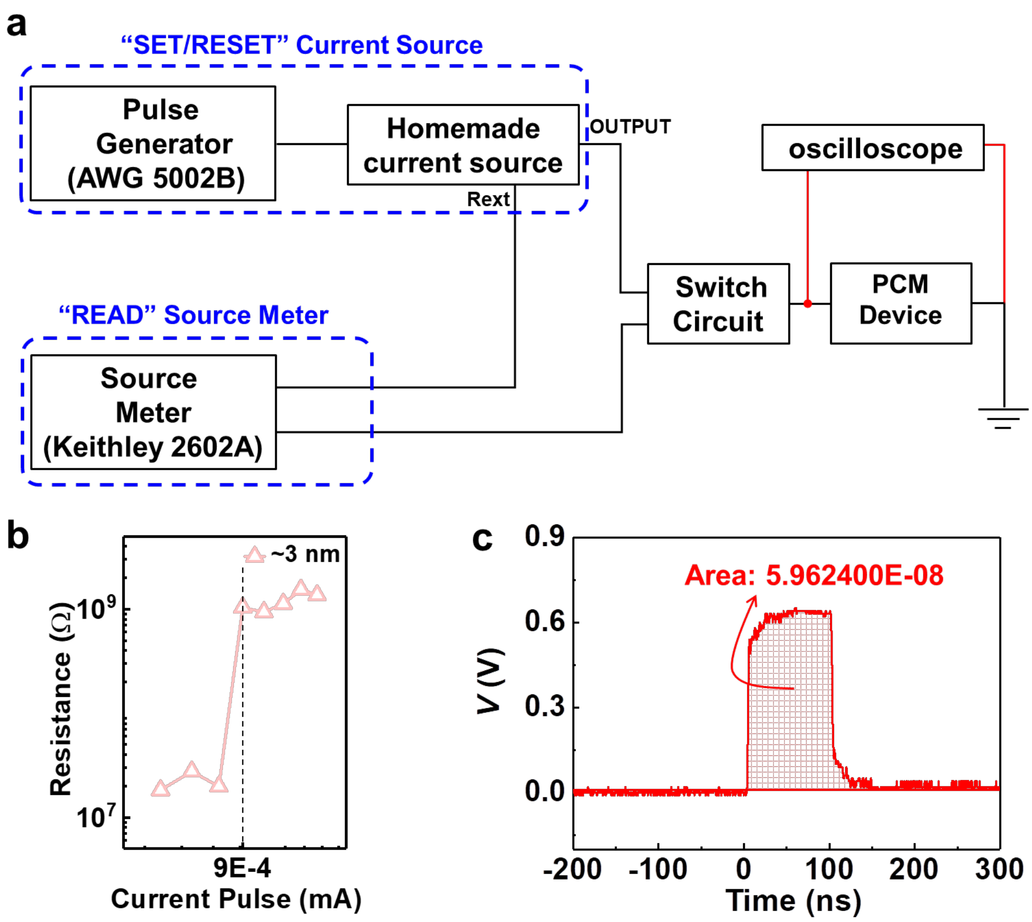

Figure S4. Power consumption measurement. a) the schematic of power consumption measurement system. **b**) Resistance versus current pulse magnitude in a memory cell with ~3 nm-wide GNR edge-contact. **c**) A captured waveform for implementing RESET operation in the memory cell with ~3 nm-wide GNR edge-contact.

We would like to introduce how the energy consumption was measured. Figure S4a shows the schematic of the setup which includes a "SET/RESET" current source and a "READ" source meter. A home-made circuit is used to generate current pulse to conduct "SET/RESET" operation. The source meter (Keithley 2602A) was used to conduct the "READ" operation. There is a switch-circuit to separate the two parts. The measurement starts from a high resistance state (amorphous state) of the memory cell. A series of current pulses with the fixed duration (100 ns) are sourced to the memory cell with the pulse amplitude gradually increased. The resistance status of the cell was recorded by the source meter (Keithley 2602A) by applying a DC voltage of 0.1V after every operation of sourcing current pulse was finished. The operation is known as "READ". "READ" and sourcing current pulse (also known as the operation of "SET/RESET") cannot be carried out simultaneously.

First, the current pulse was sourced to the memory cell by the current source shown in

Figure S4a. Then, the oscilloscope is used to capture the transient voltage (RESET voltage) of the memory cell during the RESET operation. The RESET current sourced and the waveform captured are shown in Figure S4b and S4c, respectively.

The power consumption is calculated:

$$E=\int_0^{\Delta t} IVdt = I\int_0^{\Delta t} Vdt = 9\times10^{-7}\times\int_0^{100\ \mathrm{ns}} Vdt = 5.366\times10^{-14}\ \mathrm{J}$$

Here, I represents RESET current, V is RESET voltage, and Δt is a time symbol for pulse duration. The current pulse waveform cannot be captured by an oscilloscope. Based on the waveform of voltage captured on the cell load (shown in Figure S4c), it is reasonable to believe that the current pulse is a standard square wave.

No. of round	1st	2nd	3rd	4th	5th
E (10^{-15}J)	53.66	54.17	53.72	53.69	53.25

Table S1 Measurement results for the power consumption of a memory cell with ~3 nm-wide GNR edge-contact.

The memory cell was measured for 5 times, and the results are shown in Table S1. We extract the average of these results as the power consumption for the memory cell with ~3 nm-wide GNR edge-contact.

$$\bar{E} = \frac{\sum_{i=1}^{5} E_i}{5} \approx 53.71 \times 10^{-15} J \approx 53.71\ fJ$$

The error bar of the measurement comes from standard deviation:

$$\sigma = \sqrt{\frac{\sum_{i=1}^{5}(E_i - \bar{E})^2}{5}} = 0.292$$

The power consumption of the memory cell with ~3 nm-wide GNR edge-contact is 53.7 ±0.3 fJ. In the main manuscript, it is simply recorded as ~53.7fJ.

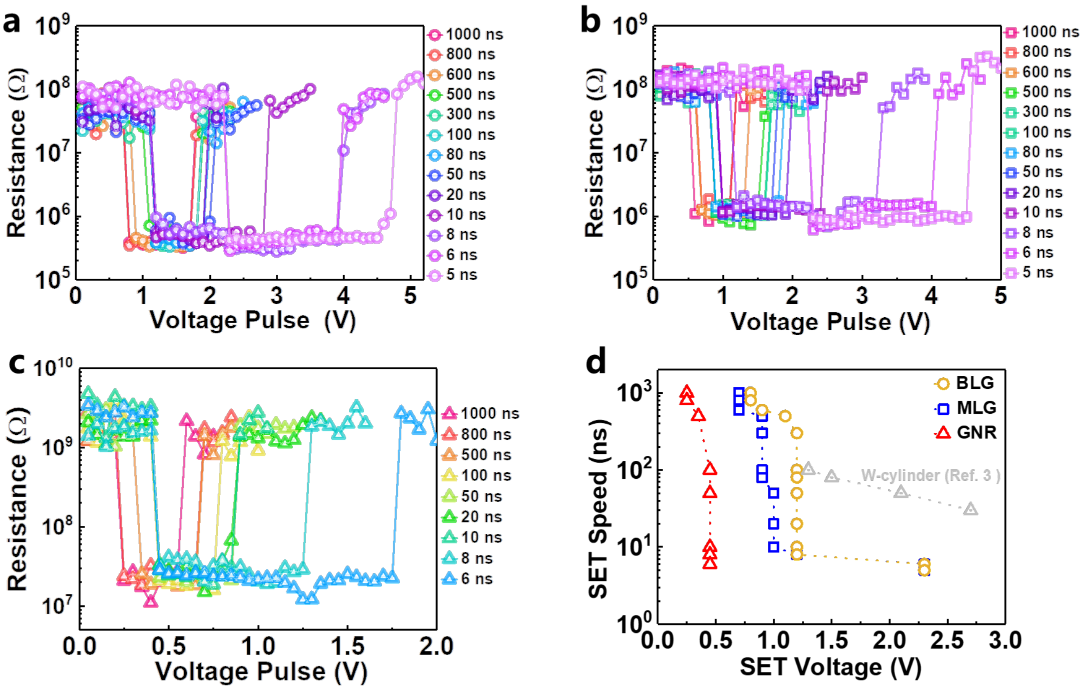

Figure S5. Resistance-voltage characteristic of memory cells. a) the cell (*#BLG 33*) with BLG edge-contact. **b**) the cell (*#MLG 51*) with MLG edge-contact. The widths of contact are ~30 nm. **c**) the cell (*#GNR 69*) with ~ 3 nm wide GNR edge-contact cell. **d**) Voltage dependence of SET speed for memory cells with edge contact. The contact widths of graphene (BLG, MLG) and GNR are ~30 nm and ~3 nm, respectively.

In addition, SET speed and cycle endurance of the memory cells with edge-contact are also examined. The memory cells are at the OFF state (HRS) in the beginning as the as-deposited GST is amorphous and highly resistive. It could be difficult to realize reversible switching in our tests. Therefore, it is necessary to set the memory cells to ON state (LRS) in the first step by annealing. Reversible switching of the cells was tested by applying voltage pulses. The relationship of resistance *versus* voltage pulse in cells with edge contact of MLG, BLG and GNR is plotted in Figure S5. It is found that both the cells can be reversibly switched by using voltage pulses in a width of 5 ns. Here, the width of pulse is also regarded as SET speed. The SET speeds of those cells are extracted from Figure S5a to S5c and the voltage dependence of SET speed are plotted in Figure S5d. Here, the minimum of voltage pulse under which the resistance of cell switches to ON state is defined as SET voltage. As shown in Figure S5d, it is found that, under the same speed, the SET voltage decreases with the width and thickness of graphene. The phenomena are attributed to reduction of contact areas. We noticed that the memory cell (*#GNR 69*) with GNR edge-contact achieves a SET speed of 6 ns at ~0.45 V, which could fulfill the demands on speed and bias of typical DRAM [2].

It is also found that the SET speed decreases with the increase of SET voltage in all the memory cells, as shown in Figure S5d. Obviously memory cells with edge-contact

exhibit merits over traditional T-shaped device [3] in aspects of both SET speed and voltage. Noticeably, the decreasing curves for edge-contact cells are in a steep slope. This means that the SET operation still can be completed without greatly increasing the amplitude of pulse voltage, when the width of voltage pulse decreases several orders. The possible reason is that the Joule heat generated is sufficient to drive a small volume of GST in cell to its crystallization temperature ~430 K even if the pulse width is greatly reduced under a similar amplitude of pulse voltage. And the cell still can switch into LRS until a critical point for the pulse width was reached. Once the critical point was reached, higher amplitude of voltage pulse is necessary for the cell to complete the SET operation. The sudden turn point corresponds to the minimum of the Joule heat required for GST crystallization.

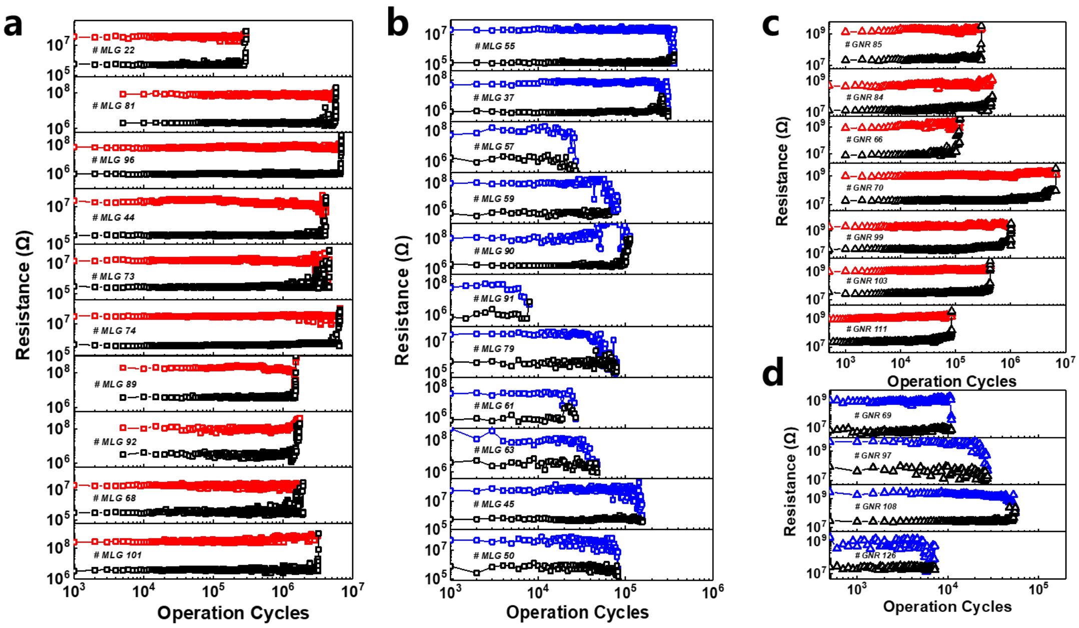

Figure S6. Data of the cells subjected to cycle endurance test. Voltage pulses in a width of 100 ns were applied from Port I to II lead to "Stuck RESET" failure in cells with **a)** MLG and **c**) GNR edge-contact, respectively. Voltage pulses in a width of 100 ns applied from Port II to I through CUTs cause that the cells with **b**) MLG and **d**) GNR edge-contact fail in "Stuck SET", respectively.

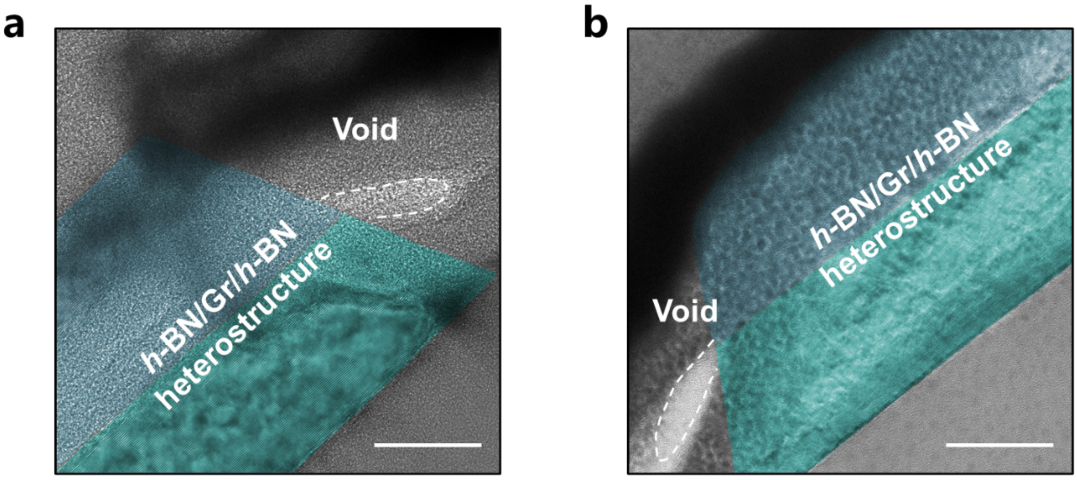

Figure S7. Cross-sectional TEM images. a) the cell No. *#MLG 101* and **b**) No. *#MLG 96* suffered from “Stuck RESET” failure after endurance test. The voids profiled by white dash line formed at the interface between graphene-edge and GST. The scale bar is 20 nm.

Figure S7 shows a cross-sectional TEM image of the cell failed in “Stuck RESET”. The white dash lines show profiles of the voids formed at the contact between graphene-edge and GST. It is believed that the formation of void causes the stay of cells at high resistance state and the rejection to SET.

We believe that the formation of void mainly comes from thermal expansion and volume change between phases in the active region of PCRAM after millions of programming operations [4].

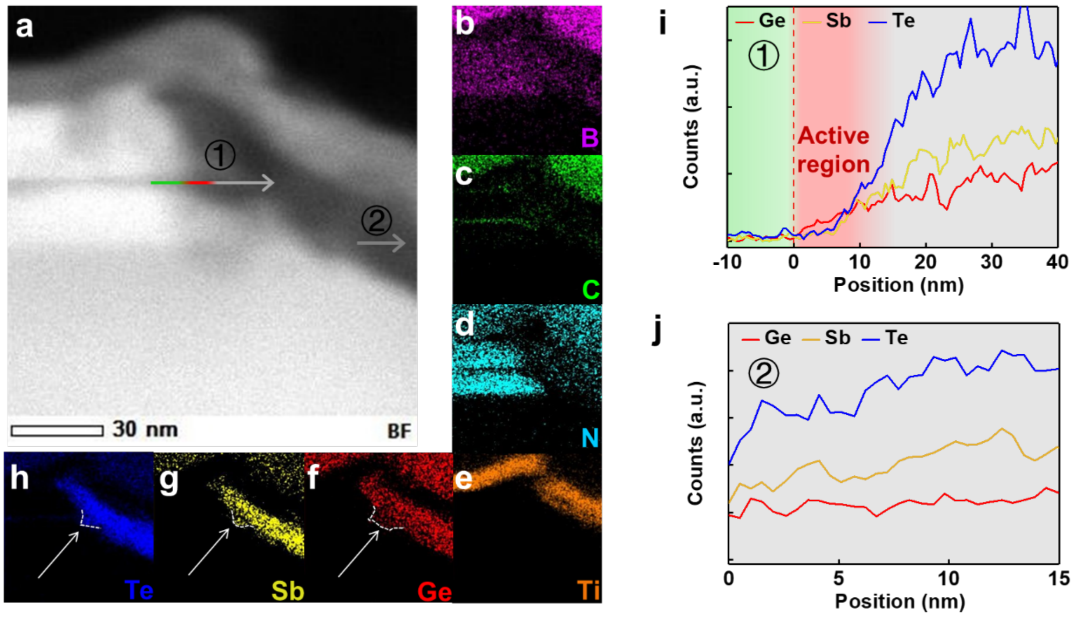

Figure S8. Cross-sectional TEM investigation on the contact region in a cell (No. *#MLG 55*) **after its failure as "Stuck SET". a**) Cross-sectional STEM image. The corresponding STEM-EDX maps for different element are shown here: **b**) Boron-B, **c**) Nitrogen-N, **d**) Carbon-C, **e**) Titanium-Ti, **f**) Germanium-Ge, **g**) Antimony-Sb, and **h**) Tellurium-Te. **i, j**) Elemental profiles of EDS line scan along line ① in active region and line ② in inactive region, respectively.

As shown in Figure S8, GST slightly intercalates into the graphene region in-between *h*-BN flakes, and Ge element is relatively rich in active region of cell. It is necessary to discuss the mechanism behind. Different from simple atomic separation induced by the electrostatic force, all the elements in molten phase moves towards graphene electrodes which is applied with negative bias. Ge and Sb atoms are likely to behave as cations and move towards graphene by electrostatic force, while Te atoms move to graphene in liquid phase by diffusion. Subsequent "incongruent melting" or "crystallization-induced segregation" [5] in the confined space of graphene between *h*-BN flakes is believed account for these atomic distribution in the active region. The phase separating behavior leads to the "SET stuck" failure after tens of thousands of programming operation.

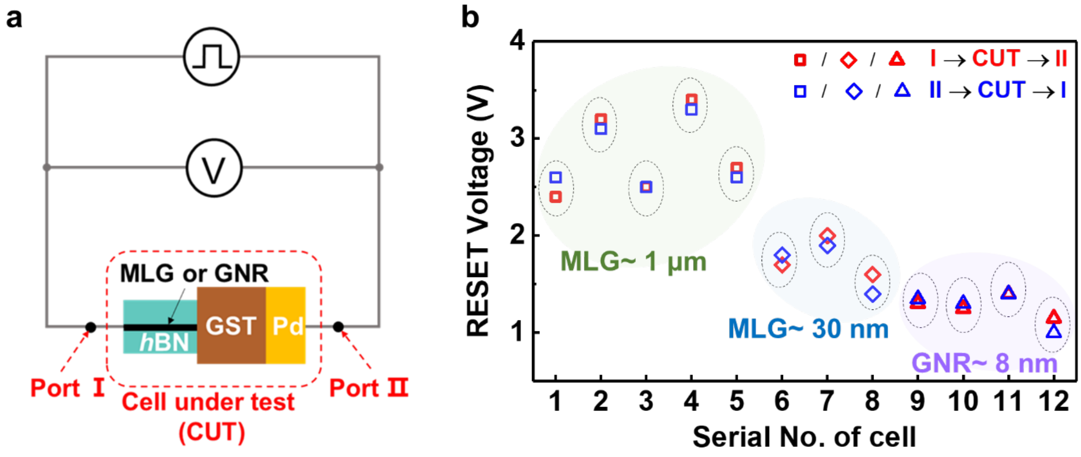

Figure S9. The influence of polarity on RESET voltage. a) Schematic of the cell and measurement layout. **b**) Statistics on the RESET Voltage for ~1 μm wide MLG, ~30 nm wide MLG and ~8 nm wide GNR edge-contact memory cells. (No.1 to 12 are corresponding to the sample *#MLG 31*, *#MLG 44*, *#MLG 50*, *#MLG 76*, *#MLG 79*, *#MLG 62*, *#MLG 91*, *#MLG 94*, *#GNR 83*, *#GNR 86*, *#GNR 97*, *#GNR 102*, respectively.)

Thermoelectric properties (Seebeck coefficient and Thomson coefficient) may be one important factor for memory cells because thermoelectric heating can significantly influence the temperature distribution in the memory cells. [6] When the current flows from graphene to GST, Peltier heating may reduce the RESET current under certain polarity. [7, 8] While Thomson heating may alter the temperature profile and the resulting shape of amorphous phase in GST material itself. [9] Both of the effects may reduce the RESET power consumption under certain polarity if thermoelectric properties play an important role. A relatively small power consumption reduces damage to memory cell, and benefit for the improvement of the cycle endurance.

Actually, it is possible to qualitatively achieve some signature of the thermoelectric effects by comparing the difference of RESET voltage under different polarity in the same device. According to the discussion in previous paragraphs, the Peltier heating that is favorable for the memory cell programming under certain polarity in the same device. Obviously, RESET voltage under different polarity should be significantly different if the thermoelectric effects play an important role.

In order to compare the effect of the voltage polarity on the RESET voltage, we carried out the statistical analysis on the voltage amplitudes of voltage pulse of different polarity in the same memory cell without taking cycle endurance measurements in those devices. As shown in Figure S9b, little difference was observed in the RESET voltages of two polarities. It indicates that the thermoelectric heating may not be pronounced during programming in the cells with graphene.

The thermoelectric heating may exist during programming in conventional PCM cells,

and it is normally a supplement to Joule heating. An unprecedentedly a high energy-efficiency of memory cell will be expected, if the majority of the heat is supplied by the thermoelectric effect. Optimizing the design of the materials makes thermoelectric heat produced during the operation of nanometer scaled memory cells crucial to rapidly melting the PCM material and switching it to an amorphous phase. We also think that thermoelectric effects may play an important role in re-designed memory cells with GNR edge contact in the future.

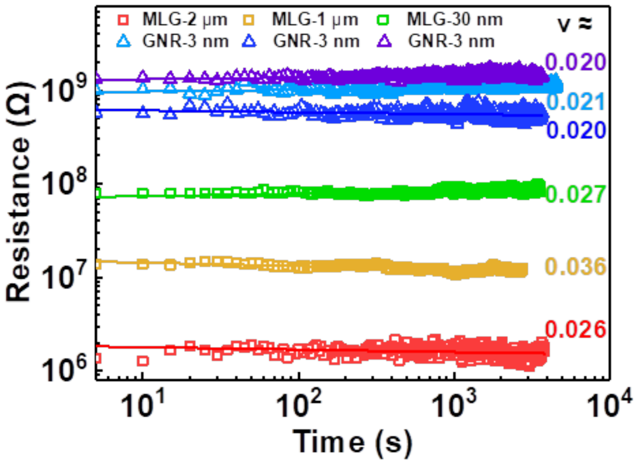

Figure S10. Resistance drift investigation. Cell resistance as a function of time for the RESET state of some edge-contacted devices. Cells with both GNR and MLG edge-contacts are tested. The amorphous state of cells with MLG and GNR edge-contact were achieved by applying voltage pulses of 100 ns. The cell resistance at amorphous state were monitored at room temperature over 1 hour with a read-voltage of 0.1 V.

The resistance drift shows the steady variation in electrical resistance of amorphous GST with time at ambient temperatures. It is regarded as a critical index in evaluation whether a material is suitable for multilevel storage. And it is strongly related to thermal/structural relaxation in amorphous GST. As shown in Figure S10, the square and triangle dots represent the experimental value of resistance drift of the cells with MLG and GNR edge-contact, respectively.

And the resistance drift is roughly depicted as: $R(\mathrm{t}) = \mathrm{abs}\ (R_0\ (t/t_0)^{v})$, where R_0 is the initial resistance at t_0, and v represents the fitted resistance drift coefficient. The power-law exponents (v) are fitted to be 0.02~0.03 in the cells. It is significantly smaller than that of a normal memory cell with cylinder electrode (*v=~0.1*) [10], while similar to that of the memory cell with insert projection layer in-between GST and the cylinder electrode (*v=~0.02*) [11].

In order to explore the main possible reason for the small drift of resistance, we fabricated some memory cells addressed with graphene edge contact. The difference is that *h*-BN multilayers were damaged by N_2 plasma (in the power of 30 W) before assembling vdW heterostuctures. The plasma treatment breaks the covalence bonds and degrades the in-plane thermal conductivity. We name the *h*-BN flakes subjected in N_2 plasma for 30 seconds as “highly defective *h*-BN”, and name the *h*-BN flakes treated in N_2 plasma for 3 minutes as “amorphous *h*-BN”. We also replace the bottom *h*-BN substrate to SiO_2, SiC crystal and diamond crystal, respectively. The substrates have different thermal conductivity (~1.2 W/m·K for SiO_2 film, 400W/ m·K for *h*-BN (in plane direction), 2W/ m·K for *h*-BN (out of plane direction), ~347 W/ m·K for SiC (0001) substrate and ~2000 W/ m·K for diamond (001) substrate). All the measurement results are summarized in Table S2.

Type	Configuration	Drift coefficient	cycles	Operation speed	RESET current
**I**	*h*-BN/MLG/*h*-BN	0.036	3.2E6	5 ns@2.3 V	0.45 mA @100 ns
**II**	Highly defective *h*-BN/MLG/ highly defective *h*-BN	0.087	2.6E4	50 ns@1.2 V	0.4 mA @100 ns
**III**	Amorphous *h*-BN/MLG/amorphous *h*-BN	0.103	1.1E4	80 ns@2.0 V	0.45 mA @100 ns
**IV**	*h*-BN/MLG/SiO_2	0.052	4.7E4	30 ns@1.7 V	0.4 mA @100 ns
**V**	Highly defective *h*-BN/MLG/SiO_2	0.101	8.0E3	100 ns@1.8 V	0.45 mA @100 ns
**VI**	Amorphous *h*-BN/MLG/SiO_2	0.131	2.1E3	100 ns@1.2 V	0.5 mA @100 ns
**VII**	*h*-BN/MLG/SiC(0001)	0.020	6.6E5	10 ns@3.4V	0.7 mA @100 ns
**VIII**	*h*-BN/MLG/diamond(001)	0.016	5.3E2	20 ns@4.1 V	2.1 mA @100 ns

Table S2. The performance of phase change memory cells with different configurations. All the edge contacts have the same width of ~1 μm.

As shown in Table S2, Type I configuration is based on the heterostructure of *h*-BN/MLG/*h*-BN, MLG is perfectly protected by high quality *h*-BN, enabling memory cells with a resistance drift coefficient of ~0.036. The resistance drift coefficient of the highly defective *h*-BN/MLG/ highly defective *h*-BN based memory cell and amorphous *h*-BN/MLG/amorphous *h*-BN based phase change memory cell is ~0.087 and ~0.103, respectively. The plasma treatment breaks the covalence bonds in *h*-BN and degrades/destroyed the in-plane thermal conductivity of *h*-BN flakes. It is obvious that resistance drifts are related to the crystalline quality of *h*-BN flakes.

In order to investigate the relationship of resistance drift and the thermal conductivity further, the bottom *h*-BN flakes are replaced by SiO_2 layer (thermal conductivity ~1.2 W/m·K for SiO_2 film). High quality *h*-BN, highly defective *h*-BN and amorphous *h*-BN are used as capping layer. We also fabricated memory cells with *h*-BN/MLG/SiO_2 edge contact (type IV), highly defective *h*-BN/MLG/SiO_2 edge contact (type V) and amorphous *h*-BN/MLG/SiO_2 edge contact (type VI). The resistance drift coefficient of the type IV cell is ~0.052. The resistance drift coefficient increase significantly if *h*-BN flakes treated with plasma are adapted (type V: ~0.101, type VI: ~1.131).

Finally, the substrates with high thermal conductivity (~347 W/ m·K for SiC (0001) substrate and ~2000 W/ m·K for diamond (001)) are used. The phase change memory cells based on type VII(*h*-BN/MLG/SiC(0001)) and type VIII(*h*-BN/MLG/diamond(001)) have a smaller resistance drift coefficient. The high thermal conductivity of SiC and diamond is beneficial to improve the resistance stability of amorphous GST.

It can be seen from the Table S2, a high thermal conductivity of dielectric substrate benefits for a low drift coefficient and a high switching speed to some extent. However, it may also lead to an increase of power consumption, which is reflected in the increase of RESET current. The excess Joule heat may cause the damage of interface between PCM(GST) and MLG-electrode, and finally shortens the cycle endurance of memory cells. As such, creating the balance between the resistance drift and the power consumption is the key for design of memory cells.

In order to investigate the influence of falling time on the resistance drift, we extended the falling time of voltage pulse to 3 ns, 4 ns, 5 ns and 6 ns, respectively. However, we did not find obvious dependence of resistance drift on the falling time. A longer falling time than 6 ns always spoil the amorphous state of the device. In this case, it looks difficult to achieve a conclusive result about the influence of falling time of voltage pulse on resistance drift. On the other hand, our results indeed show that the devices fabricated on substrates with high thermal conductivity always exhibit a low resistance drift. This is contrary to the common knowledge. It is indeed an interesting issue which is really worth further investigation.

In our opinion, there are two possible reasons of the small resistance drift for our case: (1) edge contact of graphene reduces greatly the volume of active region, and the small active region normally leads to less stresses together with the high thermal conductivity of graphene and *h*-BN. (2) The weak van der Waals interactions between GST and *h*-BN or GNR could cause full relaxation of amorphous GST [1]. In contrast, the defective *h*-BN or SiO_2 results in more reactive bonds whose reaction and relaxation may lead to a higher resistance drift.

a

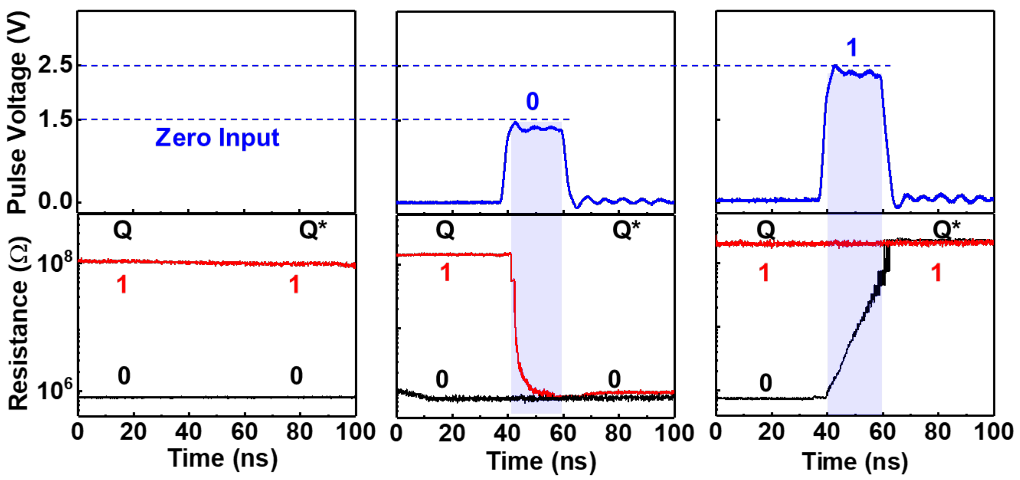

b

D	Q	Q*
Zero Input		Q
0 (1.5V)	0	0
0	1	0
1 (2.5 V)	0	1
1	1	1

c

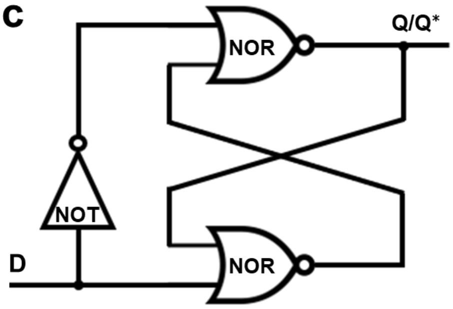

Figure S11. The prototypical D latch made from a memory cell with graphene edge-contact. a) Logic function demonstration of the D latch. **b**) the truth table of D-type latch. **c**) Logic diagram (composed of NOT and NOR gates) for the D-type latch.

As the nonvolatile memory cells can perform the function of resistive switching, we demonstrated the logic function of D latch based on the memory cells with edge-contact. The results are shown in Figure S11. Figure S11a displays the logic function of D latch. Input signals can modify the magnitude of output resistance. It is always expressed as the switching of logic states in the cell. Here, "D", "Q", and "Q*" denote input, initial state, and subsequent state, respectively. Both pulse of high voltage (2.5 V) and HRS are defined as logical "1", while pulse of low voltage (1.5 V) and LRS are defined as logical "0". As shown in Figure S11a, if no voltage pulse is applied (D = "zero input"), the subsequent state (Q*) remains the initial state (Q). When a voltage pulse in 1.5 V (D = logical "0") is sourced, the cell switches from the HRS to LRS. Similarly, a voltage pulse of higher magnitude (2.5 V) (D = logical "1") will switch the cell from the LRS to HRS. The voltage for reading the cell resistance always remains less than 0.1 V unless otherwise noted. The corresponding truth table and logic diagram are shown in Figure S11b and S11c, respectively.

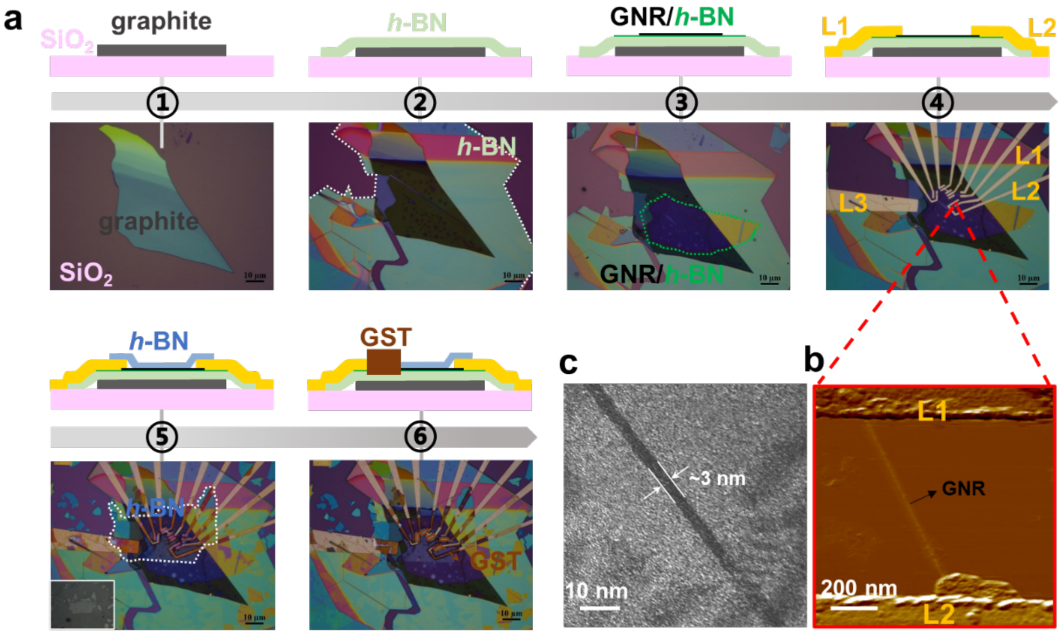

Figure S12. Fabrication for D flip-flop with edge-contact of GNR. a) the fabrication processes. **b**) The AFM image of GNR after depositing metal electrodes. **c**) The TEM image of GNR with ~3 nm-wide.

As shown in Figure S12a, firstly, a flake of graphite is peeled onto the silicon substrate with 300 nm SiO_2 to serve as the back-gate electrode of the device. After that, *h*-BN, which is ~52 nm thick, is then transferred onto the graphite. Subsequently, a flake of *h*-BN with GNRs was stacked on the *h*-BN, as shown the in ③ in Figure S12a. The GNRs are then contacted with Pd (10 nm) leads (L1 and L2) defined by lithographic patterning. Meanwhile, the Pd lead (L3) contacting back-gate graphite was deposited. We can find GNR by AFM, and the AFM image of GNR is shown in Figure S12b. After that, a flake of *h*-BN with ~20 nm-thick covers the GNR devices and acts as a protective layer. A window between metal lead and GNR was opened via RIE etching and then the ~30 nm-thick film of GST was deposited into it. After annealing at 260 °C, the memory devices based on GNRs are ready for electrical measurement. A TEM image of the ~3 nm-wide GNR is shown Figure S12c.

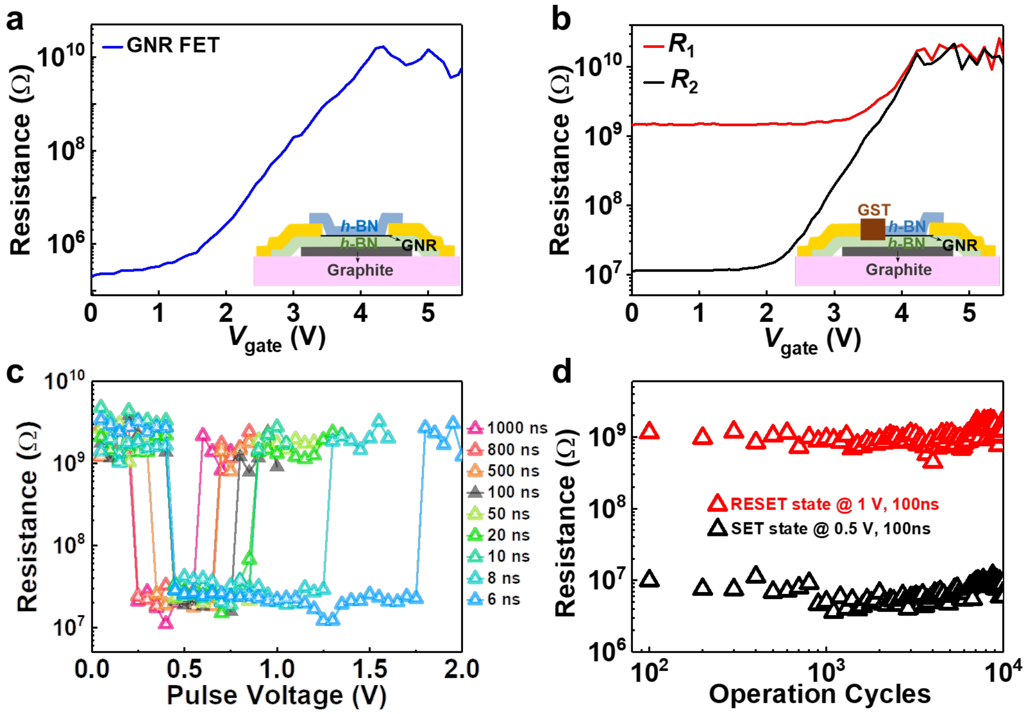

Figure S13. Operation of a D flip-flop made from a memory cell with GNR edge-contact. a) The resistance as the function of V_{gate} in the GNR FET with ~3 nm width. **b**) The resistance versus V_{gate} at 300 K after depositing GST by sputter. **c**) The resistance-voltage characteristic window. **d**) Cycle endurance. SET (0.5 V)/ RESET (1 V) with 100 ns-width of pulse voltage.

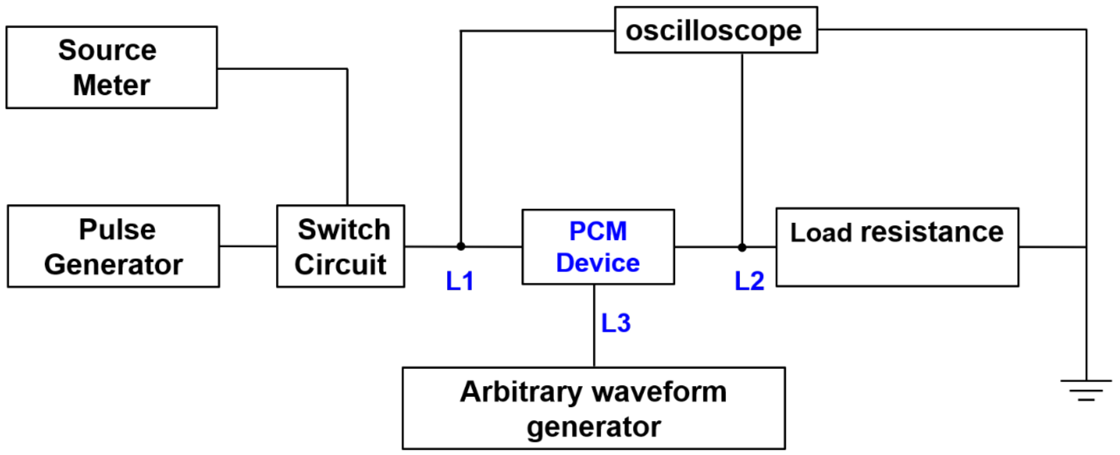

Figure S14. Schematic of the electrical test setup.

The pulse generator produces voltage pulse to induce phase change in memory device. The source meter records the change of the device resistance. The arbitrary waveform generator provides CLK signal. A constant resistance about 10^8 Ω is connected in series. The oscilloscope monitors the variation of waveforms in the memory devices and the load resistance. the change of current through the PCRAM device can be deduced by monitoring the change of voltage in load resistance.

a

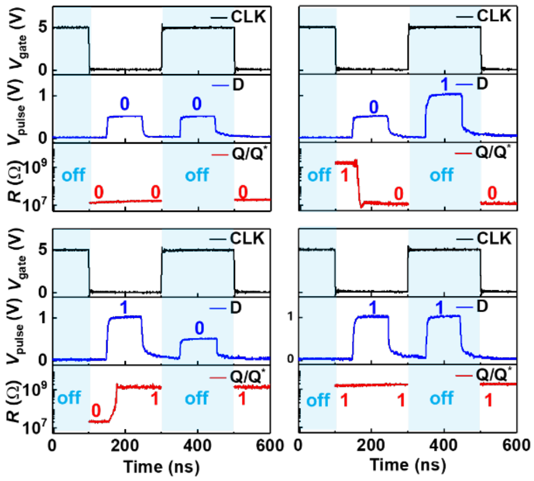

b

CLK	FET	Input pulse	D	Q	Q*
1 (0 V)	on	0.5 V	0	0 (R_L)	0
1	on	0.5 V	0	1 (R_H)	0
1	on	1 V	1	0	1
1	on	1 V	1	1	1
0 (5 V)	off	0.5 V	0	0	0
0	off	0.5 V	0	1	1
0	off	1 V	1	0	0
0	off	1 V	1	1	1

Figure S15. Details about logic functions in the D flip-flop made from a PCRAM cell with GNR edge contact. a) Logic function demonstration of the prototype D flip-flop device. **b**) The truth table of the device.

Figure S15 shows a dynamic logic function of the D flip-flop under a clock (CLK) signal of 2.5 MHz. We set 0.5 V/1 V as SET/RESET voltage with a pulse width of 100 ns to complete the reversible switch from HRS (~10^9 Ω) to LRS (~10^7 Ω) (Figure S13D). The input D in a voltage pulse of 0.5 V (1V) is regarded as logical "0" ("1"). Q and Q* represent the initial and subsequent resistance state of the memory cell, respectively. The LRS of cell means logical "0" while HRS in cell is considered as logical "1". When CLK is in 0 V (logical "1"), Q* becomes logical "0" or "1" regardless of the initial resistance state, by following D. However, when CLK is in 5 V, Q* will not change but remains in its initial state of Q. The transient change of resistance can be measured directly by an oscilloscope, the details about measurement is shown in Figure S15a. It is noted that the switch between two states takes ~20 ns, greatly shorter than 100 ns of input pulse. This indicates that the excrescent electrical energy converts to the Joule heat without causing amorphous–crystalline transitions. The corresponding truth table is shown in Figure S15b. D flip-flop normally takes lots of transistors to implement in the traditional CMOS technology. Here, the memory cell with GNR edge-contact realized the logic function of a D flip-flop, which just is composed of PCMs and GNR channel. In addition, the D flipflop is of the capability of nonvolatile memory. It could be used to produce low power arithmetic/logic units in nonvolatile random-access memories.

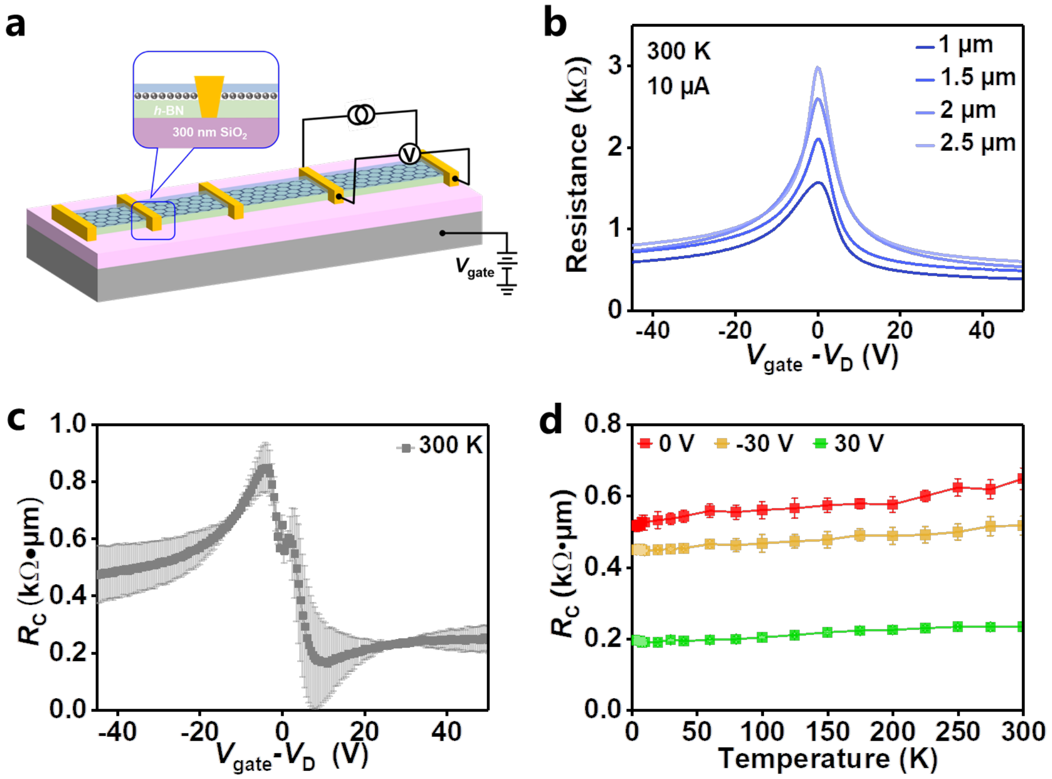

Figure S16. Resistivity of edge-contacts of graphene to metal Pd in a *h*-BN/monolayer graphene/*h*-BN hetero-structure. a) The schematic of the hetero-structure device. **b**) The resistance as a function of $\Delta V = V_{gate} - V_D$ with the different channel length in 10 μA at 300 K. **c**) Contact resistance as a function of ΔV at 300K. **d**) Temperature dependence of contact resistance at different ΔV.

Figure S16a shows the schematic of the devices. We use the transfer-length method (TLM) to extract the resistance of the edge-contact. The graphene devices in various channel lengths and the same channel width of 2 μm, are fabricated. The metal leads (Pd) are deposited by sputter. The resistances with different channel length are measured as a function of back gate voltage, the results are plotted in Figure S16b. It is found that the longer the channel length, the larger the total resistance R for the graphene device. And the total resistance in each channel can be written as $R = 2R_c(\mathrm{W}) + \rho L/W$, where R_c is the contact resistance, L is the device length, W is the device width, and ρ is the 2D channel resistivity. The contact resistance was extracted by TLM, and the relationship between R_c and ΔV was shown in Figure S16c. It is found that the R_c is ~600 Ω·μm when V_{gate} = 0 V at 300 K. Figure S16d shows the temperature dependence of R_c at different ΔV (0 V, ± 30 V). It is found that the R_c between Pd and graphene varies a little under different temperature.

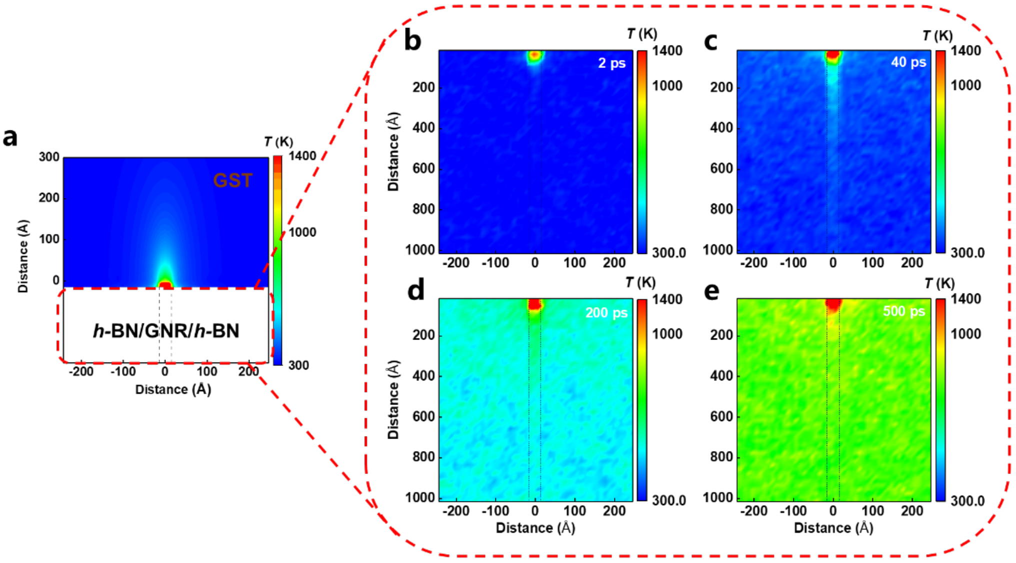

Figure S17. Modeling calculation of heat distribution. a) A comprehensive 3D finite element model for the heat distribution in GST with the initial temperature of 1400 K. The classical molecular dynamics simulation of heat transport in the graphene and *h*-BN two-dimension materials, and the initial temperature is set at 1400 K. The heat distribution at **b**) 2 ps, **c**) 40 ps, **d**) 200 ps and **e**) 500 ps, respectively.

As shown in Figure S17a, a comprehensive 3D finite element model is used to analyze the heat distribution in GST with the initial temperature of 1400 K to perform a RESET operation. The heat distribution in GST is related to its density, conductivity and the heat capacity at constant pressure. The 3D finite element model uses the following parameters of GST in crystalline state to capture the temperature distribution: density - 6200 kg/m^3, thermal conductivity - 0.46W/m/K and the heat capacity at constant pressure - 202 J/kg/K.

In order to investigate the heat transport in the graphene and *h*-BN multidimensional heterostructure, we carried out classical molecular dynamics simulations to calculate the lattice-vibration-induced heat transport, which is performed by the LAMMPS software [13]. The interatomic potential [14] is used to describe the interatomic interactions, which was developed to study the thermal transport in the graphene/*h*-BN interfaces. A 50 nm × 200 nm model with 368000 atoms is constructed. A 3 nm × 6 nm region is considered as heat source, whose temperature is set at 1400 K and is controlled by the NVT) ensemble using the Nose-Hoover thermostat, i.e. constant particle number, volume, and temperature. The other region is implemented by a NVE ensemble of the initial temperature at 300 K, i.e. constant particle number, volume, and enthalpy. In the simulations, the timestep is set as 2 fs.
The self-heating of GNR electrode under an applied pulse current with an amplitude of 0.9 μA with 100 ns was also calculated. The temperature change caused by self-heating is weak, and the overall temperature increase does not exceed 10 K after 500 ps. The heat distribution in the graphene and *h*-BN multidimensional heterostructure at different times were shown in Figure S17b to S17e.

Memory type	Ref. No.	Structure	Speed/Pulse magnitude	Power consumption for a cell		Cycles
				Energy	Parameters of electrical pulse	
PCRAM	This work	Pd-GST-GNR (edge contact)	6 ns/0.45 V	~53.7 fJ	0.9 µA/100 ns	3×10^5
	Ref.[15]	GST was deposited in CNT gap (side contact together with end contact)	150 ns/1.5 µA	~100 fJ	>1.5 µA /20 ns	2×10^2
	Ref.[16]	GST was deposited in CNT gap (side contact together with end contact)	300 ns/0.1 µA	~80 fJ	1.6 µA/100 ns	10^3
	Ref.[17]	GST was deposited in CNT gap (side contact together with end contact)	50 ns/0.5 µA	210 fJ	1.4 µA/50 ns	10^2
	Ref.[18]	GST was deposited in graphene ribbon gap (side contact together with edge contact)	N. A.	N. A.	< 10 µA/100 ns	12
	Ref.[3]	TiN/GST/W (cylinder)	30 ns/2.7 V	2.1 nJ	2.1 mA/1000 ns	10^6
		TiN/PCH($TiTe_2$/Sb_2Te_3)/W (cylinder)	8 ns/1.5 V	0.27 nJ	0.9 mA/1000 ns	2×10^9
	Ref.[19]	TiW/GST/TiW (porelike)	500 ps/0.95 V	N. A.		10^4
	Ref.[20]	TiN/GST/Sb_2Te/W (cylinder)	20 ns/0.8 V	N. A.		10^5
	Ref.[21]	TiN/CGST/TiN (blade)	100 ns/0.1 mA	N. A.	0.6 mA/100 ns	$>10^8$
	Ref.[22]	TiN/CGST/TiN (blade)	20 ns/0.1 mA	N. A.	0.64 mA/50 ns	10^8
	Ref.[23]	TiN/$Sc_{0.2}Sb_2Te_3$/W (cylinder)	700 ps/5.3 V	0.56 nJ	0.4 mA/1000 ns	10^5
	Ref.[24]	TiN/$Ti_{0.43}Sb_2Te_3$/W (cylinder)	6 ns/1.3 V	0.95 nJ	0.5 mA/1000 ns	10^7
RRAM	Ref.[25]	Ag/a-SVO/Pt	100 ns	N. A.		10^2
	Ref.[26]	TiN-HfOx-graphene	500 ns	~63 pJ	5 µA/500 ns	10^3
	Ref.[27]	TiN-HfOx-graphene	N. A.	230 fJ	2.3 µA /500 ns	$>1.6\times10^3$
Flash memory	Ref.[28]	MoS_2/*h*-BN/graphene	20 ns/30 V	N. A.		$>1.39\times10^3$
	Ref.[29]	InSe/*h*-BN/graphene	21 ns/20.2 V	N. A.		2×10^3

	Ref.[30]	BP/graphene/*h*-BN/graphene	0.3 ms/20 V	N. A.	tens

Table S3. Summary of key parameters and performance of representative non-volatile memory.

PCRAM, resistive random-access memory (RRAM) and Flash memory are all recognized as important technologies capable of storing information in a non-volatile way. Power consumption, operating speed and cycle endurance are key parameters to evaluate the performance of non-volatile memory. We present in detail a statistical analysis of the reported performance of representative non-volatile memory in Supplementary Table S3. As shown in Supplementary Table S3, the PCRAM cell with ~3 nm-wide GNR as edge-contact presented in this work exhibits a power consumption about 53.7 fJ, which is the lowest among all PCRAM cells reported. In addition, the PCRAM cell exhibits among the best combination of an operating speed (~6 ns) and a cycle endurance (~3×10^5) in all non-volatile memories shown in Table S3. Because GNRs serve as both edge-contact electrode and channel material, a low power arithmetic/logic unit can be built-up in simplified device structure.

References:

[1] Y. Wakafuji, R. Moriya, S. Masubuchi, K. Watanabe, T. Taniguchi, T. Machida, *Nano Lett.* **2020**, *20*, 2486.

[2] W. Banerjee, *Electronics* **2020**, *9*, 1029.

[3] K. Ding, J. Wang, Y. Zhou, H. Tian, L. Lu, R. Mazzarello, C. Jia, W. Zhang, F. Rao, E. Ma, *Science* **2019**, *366*, 210.

[4] S. H. Oh, K. Baek, S. K. Son, K. Song, J. W. Oh, S.-J. Jeon, W. Kim, J. H. Yoo, K. J. Lee, *Nanoscale Adv.* **2020**, *2*, 3841.

[5] A. Padilla, G. W. Burr, C. T. Rettner, T. Topuria, P. M. Rice, B. Jackson, K. Virwani, A. J. Kellock, D. Dupouy, A. Debunne, R. M. Shelby, K. Gopalakrishnan, R. S. Shenoy, B. N. Kurdi, *J. Appl. Phys.* **2011**, *110*, 054501.

[6] J. Lee, M. Asheghi, K. E. Goodson, *Nanotechnology* **2012**, *23*, 205201.

[7] G. Skoblin, J. Sun, A. Yurgens, *Sci. Rep.* **2017**, *7*, 15542.

[8] J. Lee, T. Kodama, Y. Won, M. Asheghi, K. E. Goodson, *in ASME 2012 Third International Conference on Micro/Nanoscale Heat and Mass Transfer*, **2012**, 687.

[9] A. Faraclas, G. Bakan, L. H. Adnane, F. Dirisaglik, N. E. Williams, A. Gokirmak, H. Silva, *IEEE Transactions on Electron Devices* **2014**, *61*, 372.

[10] I. Boybat, S. R. Nandakumar, M. Le Gallo, B. Rajendran, Y. Leblebici, A. Sebastian, E. Eleftheriou, *Proc. Non-Volatile Memory Technol. Symp.* **2018**, 1.

[11] R. L. Bruce, S. G. Sarwat, I. Boybat, C. W. Cheng, W. Kim, S. R. Nandakumar, C. Mackin, T. Philip, Z. Liu, K. Brew, N. Gong, I. Ok, P. Adusumilli, K. Spoon, S. Ambrogio, B. Kersting, T. Bohnstingl, M. L. Gallo, A. Simon, N. Li, I. Saraf, J. P. Han, L. Gignac, J. M. Papalia, T. Yamashita, N. Saulnier, G. W. Burr, H. Tsai, A. Sebastian, V. Narayanan, M. BrightSky, *IEEE Int. Reliab. Phys. Symp. Proc.* **2021**, 1.

[12] X. P. Wang, X. B. Li, N. K. Chen, B. Chen, F. Rao, S. Zhang, *Adv. Sci.* **2021**, *8*, 2004185.

[13] S. Plimpton, J. *Comput. Phys.* **1995**, *117*, 1.

[14] A. Kınacı, J. B. Haskins, C. Sevik, T. Çağın, *Phys. Rev. B* **2012**, *86*, 115410.

[15] F. Xiong, A. D. Liao, D. Estrada, E. Pop, *Science* **2011**, *332*, 568.

[16] F. Xiong, M. H. Bae, Y. Dai, A. D. Liao, A. Behnam, E. A. Carrion, S. Hong, D. Ielmini, E. Pop, *Nano Lett.* **2013**, *13*, 464.

[17] J. Liang, R. G. D. Jeyasingh, H.-Y. Chen, H. S. P. Wong, *IEEE Trans. Electron Dev.* **2012**, *59*, 1155.

[18] A. Behnam, F. Xiong, A. Cappelli, N. C. Wang, E. A. Carrion, S. Hong, Y. Dai, A. S. Lyons, E. K. Chow, E. Piccinini, C. Jacoboni, E. Pop, *Appl. Phys. Lett.* **2015**, *107*, 123508.

[19] D. Loke, T. H. Lee, W. J. Wang, L. P. Shi, R. Zhao, Y. C. Yeo, T. C. Chong, S. R. Elliott, *Science* **2012**, *336*, 1566.

[20] G. Y. Liu, L. C. Wu, S. F. Zhang, W. L. Liu, S. L. Lv, M. Zhu, S. N. Song, Z. T. Song, *Appl. Phys. Lett.* **2019**, *115*, 133505.

[21] T. Li, J. Shen, L. Wu, Z. Song, S. Lv, D. Cai, S. Zhang, T. Guo, S. Song, M. Zhu, *J. Phys. Chem. C* **2019**, *123*, 13377.

[22] Z. T. Song, D. L. Cai, X. Li, L. Wang, Y. F. Chen, H. P. Chen, Q. Wang, Y. P. Zhan, M. H. Ji, *in Proc. Int. Electron Devices Meeting*, **2018**, *27*.5.1.
[23] F. Rao, K. Ding, Y. Zhou, Y. Zheng, M. Xia, S. Lv, Z. Song, S. Feng, I. Ronneberger, R. Mazzarello, W. Zhang, E. Ma, *Science* **2017**, *358*, 1423.
[24] M. Zhu, M. Xia, F. Rao, X. Li, L. Wu, X. Ji, S. Lv, Z. Song, S. Feng, H. Sun, S. Zhang, *Nat. Commun.* **2014**, *5*, 4086.
[25] T. J. Lee, S. K. Kim, T. Y. Seong, *Sci. Rep.* **2020**, *10*, 5761.
[26] B. Alimkhanuly, J. Sohn, I.-J. Chang, S. Lee, *2D Mater. Appl.* **2021**, *5*, 55.
[27] S. Lee, J. Sohn, Z. Jiang, H. Y. Chen, H. S. Philip Wong, *Nat. Commun.* **2015**, *6*, 8407.
[28] L. Liu, C. Liu, L. Jiang, J. Li, Y. Ding, S. Wang, Y. G. Jiang, Y. B. Sun, J. Wang, S. Chen, D. W. Zhang, P. Zhou, *Nat. nanotechnol.* **2021**, *16*, 874.
[29] L. Wu, A. Wang, J. Shi, J. Yan, Z. Zhou, C. Bian, J. Ma, R. Ma, H. Liu, J. Chen, Y. Huang, W. Zhou, L. Bao, M. Ouyang, S. J. Pennycook, S. T. Pantelides, H. J. Gao, *Nat. nanotechnol.* **2021**, *16*, 882.
[30] D. Li, M. Chen, Q. Zong, Z. Zhang, *Nano Lett.* **2017**, *17*, 6353.
